# Molecular Ionization Energies from GW and Hartree–Fock Theory: Polarizability, Screening, and Self-Energy Vertex Corrections

**DOI:** 10.1021/acs.jctc.4c00795

**Published:** 2024-08-27

**Authors:** Charles H. Patterson

**Affiliations:** School of Physics, Trinity College Dublin, Dublin, D02 PN40, Ireland

## Abstract

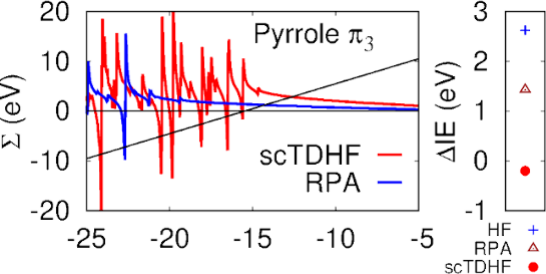

Accurate prediction of electron removal and addition energies is essential for reproducing neutral excitation spectra in molecules using Bethe–Salpeter equation methods. A Hartree–Fock starting point for *GW*/BSE calculations, combined with a random phase approximation (RPA) polarizability in the screened interaction, *W*, is well-known to overestimate neutral excitation energies. Using a Hartree–Fock starting point, we apply several different approximations for *W* to molecules in the Quest-3 database [Loos et al. *J. Chem. Theory Comput.***2020**, *16*, 1711]. *W* is calculated using polarizabilities in RPA and time-dependent HF approximations. Inclusion of screened electron–hole attraction in the polarizability yields valence ionization energies in better agreement with experimental values and ADC(3) calculations than the more commonly applied RPA polarizability. Quasiparticle weights are also in better agreement with ADC(3) values when electron–hole attraction is included in *W*. Shake-up excitations for the 1π levels in benzene and azines are indicated only when electron–hole attraction is included. Ionization energies derived from HF eigenvalues via Koopmans theorem for molecules with nitrogen or oxygen lone pairs have the largest differences from experimental values in the molecules considered, leading to incorrect ordering of nonbonding and π bonding levels. Inclusion of electron–hole attraction in the polarizability results in correct ordering of ionization energies and marked improvement in agreement with experimental data. Vertex corrections to the self-energy further improve agreement with experimental ionization energies for these localized states.

## Introduction

The quality of linear response calculations of optical properties of organic molecules depends on the accuracy of particle and hole energy levels and their interactions in the optically excited state. Performances of various density functionals,^1−5^ including hybrid functionals containing exact exchange^6−8^ and the Hartree–Fock method itself,^1,9−14^ as the starting point for *GW* calculations of particle and hole levels have been widely studied in the last 10 years or so. The *GW* approximation for particle and hole energies introduces the concept of a screened Coulomb interaction between the particle or hole and the remaining electronic charges in the system.^15^ In periodic^16^ and molecular *GW* calculations^1^ the polarizability which mediates the screened interaction is usually the polarizability in the random phase approximation (RPA). This approach is successful for predicting neutral excitations in crystalline systems^16^ where a DFT or hybrid DFT starting point is adopted. The debate about which starting point is best for calculating ionization energies (IE) and electron affinities (EA) and neutral excitations in small molecules has found that a HF starting point, combined with a screened interaction mediated by the RPA polarizability, leads to IE and neutral excitation energies which are consistently larger than experiment and accurate CCSD(T) quantum chemical calculations.^7,9−14,17−20^

Aromatic six membered rings containing one to four N atoms are essential components of current organic light emitting devices.^21−25^ They have applications as thermally activated delayed fluorescence (TADF) emitters,^23^ electron transport materials (ETM)^26^ and as sensitizers in ultraefficient hyperfluorescent light emitters.^22,25^ TADF emitters based on pyridine, pyrazine and triazine^26−29^ have been demonstrated. Electron accepting cyano groups conjugated with phenyl rings in molecules such as 2CzPN constitute an additional important class of TADF emitters.^30^

Replacement of C–H in a phenyl ring by N results in lowered HOMO (*H* – 0) and LUMO (*L* + 0) levels,^26^ with shifts increasing with the number of N in the ring. An important feature of N heterocycles is the presence of relatively localized N nonbonding, lone pair states, which form frontier orbitals. The lowest IE in pyridine, pyrazine and *s*-triazine are all associated with nonbonding states localized on N; the first two excited states of pyridine and pyrazine are *n* → π* and the first three excited states of *s*-triazine are *n* → π*.^31^ In contrast, C–H bond pairs in phenyl rings are deeper in energy than nonbonding N states; holes in these states are not important in low energy excited states. Crucially, HF theory incorrectly orders frontier levels with π and nonbonding *n* character in N heterocycles. This leads to incorrect ordering of excited states in TDHF^32^ and the need to correct HF eigenvalues in excited state calculations, for example by *GW* self-energy methods. The main source of this error is overestimation of nonbonding N orbital IE’s. Application of accurate quantum chemical methods to large molecules for photonic applications is not possible owing to their computational expense. Hence less accurate yet reliable methods are needed which yield IE and EA in acceptable agreement with experiment.

Here we present results of *GW* calculations of IE for 21 medium sized molecules in the Quest-3 database of excited state energies.^31^ Variations of *GW* self-energies which are calculated in this work depend on the form of the polarizability, Π, whether or not an electron–hole interaction is included in the polarizability (Π^*RPA*^ or Π^*TDHF*^), whether or not this is statically screened (Π^*TDHF*^ or Π^*scTDHF*^) and whether or not vertex terms in the self-energy are included.

In agreement with previous work which included the *G*_0_*W*_0_*@HF* approximation^9,12,18,20,33,34^ (which we denote Σ^*RPA*^), we find that this method overestimates IE for the 21 molecules studied here, based on the mean signed error (MSE) compared to experimental values from photoemission data. In contrast with much previous work, which has mainly focused on first IE (ionization potentials) and comparison to IE from CCSD(T) calculations, we compare up to nine IE for each molecule to experimental data. In an attempt to find an optimally tuned range-separated TDDFT method for large organic molecules, Körzdörfer et al.^33^ concluded that, ’schemes for tuning of range-separated hybrid functional have yet to achieve the accuracy of reliable *GW* methods for the calculation of quasiparticle spectra’. In particular, we find that differences, ΔIE, between experimental IE and Koopmans’ values (HF eigenvalues) for different electron pair types vary considerably. Localized, N lone pair states have ΔIE values up to 2.4 eV and a MSE of 2 eV, while the corresponding values for the highest occupied levels of π character (π_1_ levels) are 0.4 eV and −0.1 eV, respectively.

The spectral function in many-body methods consists of quasiparticle (QP) peaks with a weight approaching unity plus additional satellite peaks which result from interaction of QP with neutral excitations, etc. The spectral weight commonly appears in condensed matter physics^16^ as the solution to the QP equation using a Taylor expansion method. However, the physical significance of the QP weight (or pole strength) becomes evident in the concept of Dyson orbitals,^35^ which are overlaps of many-particle *N* and *N* ± 1 electron states. In this work we compare the QP weight from self-energy calculations to weights (pole strengths) from ADC(3) calculations,^36,37^ where available. QP weights from self-energy calculations which include the electron–hole interaction in the polarizability, Π^*TDHF*^, agree much better with ADC(3) values, especially for states where the QP weight is significantly less than unity, because of shakeup excitations. ADC(3) predicts shakeup excitations via multiple QP peaks; in the perturbative GW method used here, the existence of a shakeup state is indicated by a much reduced QP weight. The largest binding energy π states in aromatic six membered rings are good examples of such states. When an RPA polarizability is used, there is no corresponding decline in QP weight.

The remainder of this paper is structured as follows: the approximations to the self-energy which are used are described in the Theory Section the implementation of this self-energy in the Exciton code^38,39^ which uses a density fitting approach to calculate four center integrals is described in the Computational Methods Section along with details of basis sets and molecular geometries; IE from HF eigenvalues and solution of the QP equation are compared to experimental values from photoemission experiments in Section and (where available) IE and QP weights from ADC(3). A detailed comparison is given for *t*-butadiene, furan, pyrrole, thiophene and pyridine. Differences in experimental and calculated IE are presented and experimental and predicited IE are given in the Supporting Information (SI). Results are compared to previous work and conclusions regarding the best options for application of these methods to large molecules are given in the Discussion and Conclusions Section. In a related work,^32^ we will present calculations of optical excitation energies of molecules in the Quest-3 database and compare them to theoretical best estimates using the self-energies computed in this work.

## Theory

Hedin’s equations^15^ are a scheme for calculating the ingredients of a many body theory of matter, namely the interacting Green’s function, *G*, the exchange-correlation self-energy Σ_*xc*_, the screened interaction, *W* and the vertex, Γ. In principle, they are solved iteratively to convergence. Thus, far, in practice, they may be iterated to convergence with the lowest order approximation for the vertex functional, namely self-consistent *GW* (sc*GW*)^10,18,19,40−43^ or alternatively in quasi-particle self-consistent *GW* (QS*GW*).^42,44,45^ In the case of conventional *G*_0_*W*_0_,^16,17,46^ the vertex is the simplest possible, point-like truncation, δ(1, 3)δ(2, 4) in eq 5 below, while in vertex-corrected methods, the integral equation for the vertex is truncated at low order. Recent examples of this approach include the second order screened exchange (SOSEX) approach.^2,20,47,48^

Maggio and Kresse^13^ showed that it is possible to write Hedin’s equations in a four-point notation,
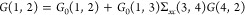
1
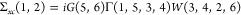
2
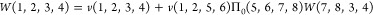
3
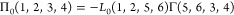
4
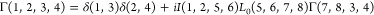
5Here, *G*_0_ is the noninteracting, HF Green’s function, *L*_0_ is the independent particle-hole propagator,
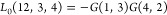
6*v* is the instantaneous Coulomb interaction,
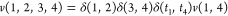
7The kernel, *I*, is,
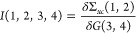
8Since *G*_0_ is the HF Green’s function, *I* is approximated as,
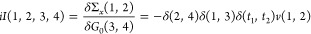
9where Σ_*x*_(1, 2) is the HF exchange operator. Maggio and Kresse^13^ showed that the product Γ*W* can be written as,
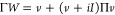
10The self-energy where the vertex in eq 10 is implicitly included is therefore,^13^
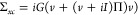
11and the polarizability is,
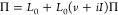
12with inverse,
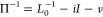
13The final, compact version of Hedin’s equations is eq 1, 11 and 12,^13^ The first term on the right in eq 11, *iGv*, is the HF exchange contribution to the self-energy. Since we adopt a HF starting point, this is already incorporated in the HF Green’s function and is omitted from the energy-dependent self-energy which is calculated here.

**Figure 1:**
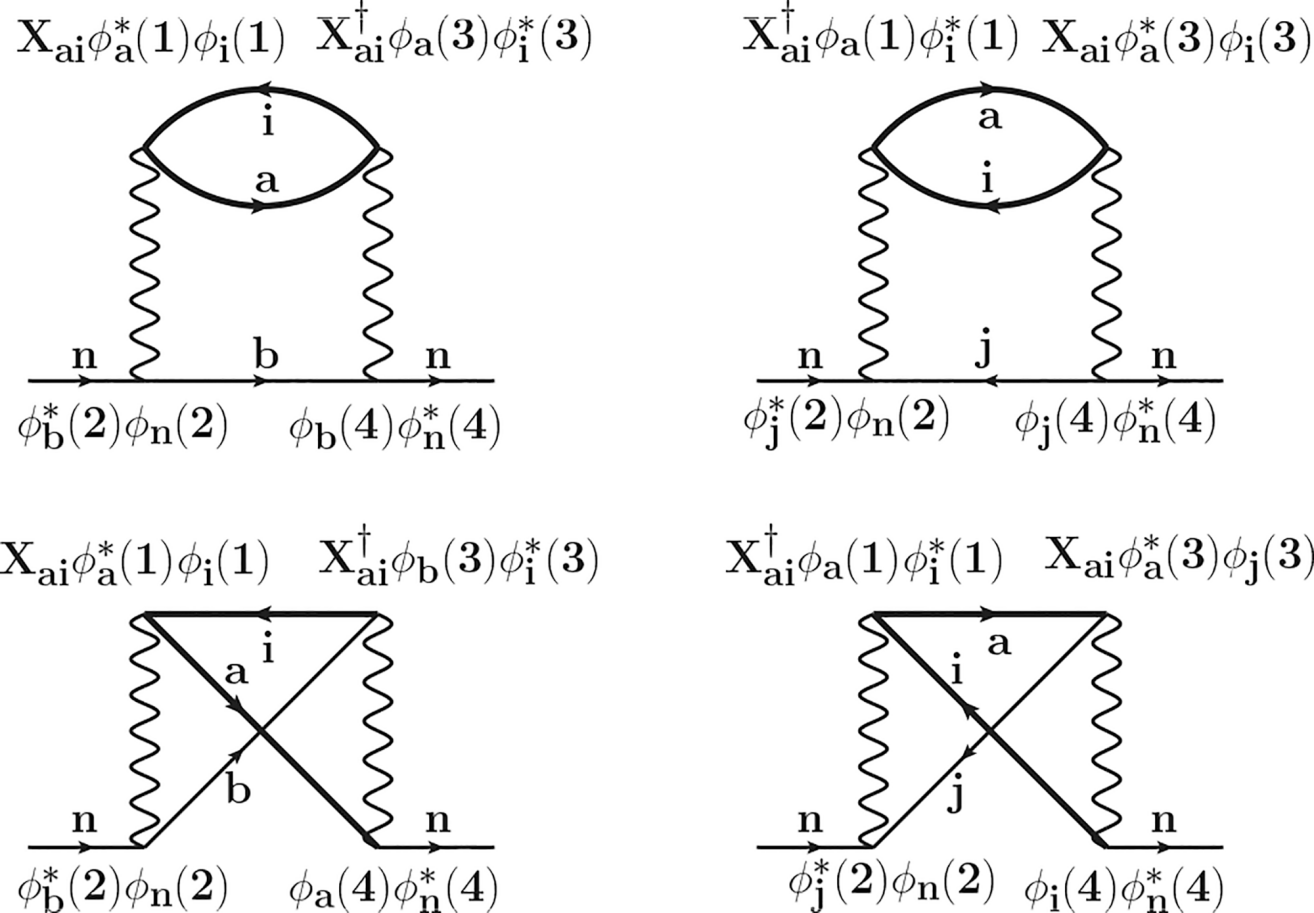
Particle and hole contributions to the self-energy of state |*n*⟩. Time order for the diagrams on the left is *t*_4_ > *t*_2_ and *t*_2_ > *t*_4_ for diagrams on the right. The top row contains the *vWv* screened interaction and the bottom row its exchange partner. Bold lines labeled *a*, *i* indicate the dressed polarizability and lines labeled *b*, *j* are *G*_o_ particle and hole lines. RPA or TDHF excitation amplitudes *X*_*ai*_ multiply densities ϕ_a_^*^ϕ_*i*_.

Note that inclusion of the kernel *I* in eq 8 leads to vertex corrections to both the polarizability (as electron–hole attraction) via eq 12 and 13 and the additional exchange self-energy diagram (eq 11) in the lower half of Figure 1.

### Polarizability

The upper and lower panels of Figure 1 illustrate the two terms in the dynamic self-energy in eq 11. The bubble with lines labeled *a* and *i* in the upper panel is a two-space point and two-time polarizability; the lines with the same labels in the lower panel are a three-space point and two-time polarizability. The polarizability is obtained in its spectral representation, beginning by solving the RPA/TDHF equation,
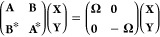
14The *A* and *B* matrices which appear in eq 14 for RPA, TDHF and scTDHF calculations with and without the Tamm-Dancoff approximation^49,50^ (TDA) are given in Table 1. There is an additional diagonal term in the *A* matrices which is (ε_*a*_ – ε_*i*_)δ_*ia*,*jb*_. Coulomb matrix elements are given in the standard ’physicist’s notation’ where ⟨*bi*|*ja*⟩=(b*j|i*a), etc. eigenvalue differences on the diagonal of the *A* matrix are *not* replaced by QP eigenvalues in scTDHF calculations.

**Table 1:** *A* and *B* Matrix Elements Used for Calculation of the Polarizability in the Π^RPA^, Π^TDHF^, and Π^scTDHF^ Approximations, with and without the TDA^a^

method	*A*_*ia*,*jb*_^*v*^	*A*_*ia*,*jb*_^*iI*^	*B*_*ia*,*jb*_^*v*^	*B*_*ia*,*jb*_^*iI*^
RPA	2⟨*bi*|*ja*⟩		2⟨*ji*|*ba*⟩	
TDHF	2⟨*bi*|*ja*⟩	–⟨*bi*|*aj*⟩	2⟨*ji*|*ba*⟩	–⟨*ji*|*ab*⟩
scTDHF	2⟨*bi*|*ja*⟩	–⟨*bi*|*W*|*aj*⟩	2⟨*ji*|*ba*⟩	–⟨*ji*|*W*|*ab*⟩
RPA-TDA	2⟨*bi*|*ja*⟩			
TDHF-TDA	2⟨*bi*|*ja*⟩	–⟨*bi*|*aj*⟩		
scTDHF-TDA	2⟨*bi*|*ja*⟩	–⟨*bi*|*W*|*aj*⟩		

a Factors of 2 appear from spin summation.

The calculation of Π^*RPA*^ from eq 14 is done without invoking the TDA. *A* and *B* matrix elements for the RPA entry in Table 1 differ by just the eigenvalue differences on the diagonal. A well-known transformation^13^ allows the full RPA matrix to be solved by diagonalizing the sum of *A* + *B* matrices. The method depends on the fact that the difference, *A* – *B*, is diagonal for calculation of the eigenvectors and eigenvalues of the full RPA matrix. The difference, *A* – *B*, is not diagonal for the TDHF matrix, and so for TDHF and scTDHF polarizabilities, the TDA has been invoked. Omission of the off-diagonal *B* blocks in eq 14 yields the corresponding TDA eigenvalue equation, *AX* = Ω*X*. The polarizabilities used in self-energy calculations are therefore as follows: Π^*RPA*^ is derived from the full RPA matrix while Π^*TDHF*^ and Π^*scTDHF*^ are derived from the TDHF-TDA and scTDHF-TDA matrices in Table 1.

Derivation of the polarizability from eq 14,^51^
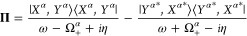
15is given in the SI. |*X*^α^,*Y*^α^⟩ is the α^*th*^ column eigenvector of eq 14 with corresponding eigenvalue, Ω_+_^α^ and ⟨*X*^α^,*Y*^α^| is the corresponding row eigenvector. Eigenvector solutions of eq 14 obey a special orthonormality condition and eigenvalues occur in positive and negative pairs with the same magnitude, denoted Ω_±_^α^. Ω_+_^α^ denotes the positive eigenvalue (see SI for more details). The *A* and *B* matrices in eq 14 are constructed in a basis of electron–hole products, ϕ_*a*_(**r**)ϕ_i_^*^(**r**) and ϕ_a_^*^(**r**)ϕ_*i*_(**r**), where ϕ_*i*_(**r**) are occupied state eigenfunctions of the Fock operator and ϕ_*a*_(**r**) are virtual states. The density response in coordinate notation is *X*_*ai*_ϕ_*a*_(**r**)ϕ_i_^*^(**r**) and *Y*_*ai*_ϕ_*i*_(**r**)ϕ_a_^*^(**r**).

For molecules with real HF orbitals and real eigenvectors, |*X*^α^,*Y*^α^⟩, all parts of the resonant and antiresonant blocks of the polarizability matrix contain products ϕ_*a*_(**r**)ϕ_*i*_(**r**). Consequently the four blocks in the polarizability matrix (see SI Eq. S11) reduce to,
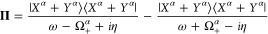
16Thus, for Π^*RPA*^ the polarizability has the form in eq 16. Since the TDA is invoked in calculations of Π^*TDHF*^ and Π^*scTDHF*^ those polarizabilities have the form,
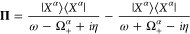
17In the absence of interactions (*A*_*ia,jb*_ = (ε_*a*_ – ε_*i*_)δ_*ia*,*jb*_ and *B* = 0), the *X* eigenvector matrix block in eq 16 becomes the unit matrix and the *Y* block becomes zero. The resulting HF polarizability in coordinate notation is,
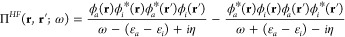
18Π^*TDHF*^ is obtained by including eigenvectors from a TDHF calculation in the polarizability,
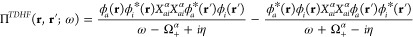
19Π^*scTDHF*^ is obtained by using eigenvectors, *X*^α^, and eigenvalues, Ω_+_^α^, from a scTDHF calculation in eq 19.

### *GW* Self-Energy Approximations

Five approximations for the self-energy are considered in this work. Σ^*RPA*^ is the conventional *G*_0_*W*_0_*@HF* self-energy. Σ^*TDHF*^ is calculated using Π^*TDHF*^ in place of Π^*RPA*^ in *G*_0_*W*_0_*@HF*, but omits the vertex correction which arises from the factor *iI* in eq 11. It corresponds to the diagrams in the top panel of Figure 1. Σ^*scTDHF*^ is calculated using Π^*scTDHF*^. When the vertex correction in the lower panel of Figure 1 is included, then the self-energy is denoted either Σ^*vTDHF*^ or Σ^*scvTDHF*^. These approximations are used to investigate the importance of electron–hole attraction and/or screening in the polarizability calculation and vertex corrections in the self-energy calculation, beginning from *G*_0_*W*_0_*@HF* where all of these are omitted.
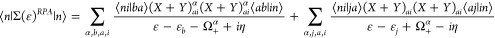
20
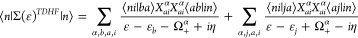
21
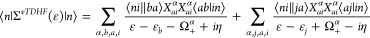
22While the TDA is applied in calculations of TDHF and scTDHF polarizabilities and self-energies defined in eq 18 to 22, a TDA superscript is omitted from Π^(*sc*)*TDHF*^ and Σ^(*sc*)*TDHF*^ for a concise notation in the text and results tables. Σ(ε)^*TDHF*^ is similar to the self-energy denoted Σ^*tc*–*tc*^ by Maggio and Kresse,^13^ where *tc* denotes a test charge. They did not apply the TDA to the polarizability calculation in that case.

### Quasi-particle Weight

Once the self-energy has been obtained, IE are obtained as QP shifts with respect to HF eigenvalues by solving the QP equation,
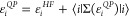
23The QP weight function is,
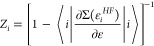
24

## Computational Methods

### Density Fitted Four Center Integrals

Calculations were performed using the Gaussian orbital Exciton code,^38,39^ which employs density-fitted electron repulsion integrals for many-body calculations on molecular^38^ and periodic systems.^39,52^ Molecular orbital products, ϕ_*i*_(1)ϕ_*a*_(1), are expanded in an auxiliary Gaussian orbital basis, χ_β_, using Coulomb weights,
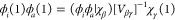
25Two and three center integrals over the Coulomb potential, *v*(1, 2), are,
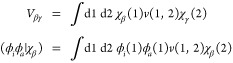
and the final expression for a four-center integral is,
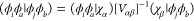


Computation of the self-energy approximations in eq 20 to 22 requires contraction of four-center integrals with eigenvectors *X*_*ai*_ or (*X* + *Y*)_*ai*_. Factorization of the four-center integrals in this way allows contraction of the factor (ϕ_*i*_ϕ_*a*_|χ_α_) with *X*_*ai*_ or (*X* + *Y*)_*ai*_, provided that both indices, *i* and *a*, are on the same integral factor. This is the case for the diagrams in the upper panel of Figure 1, but not in the vertex correction diagrams in the lower panel. Integral factors, (ϕ_*i*_ϕ_*a*_|χ_α_), are distributed over cores using the atomic center, α, on which auxiliary basis functions, χ_α_, are located. This makes it possbile to treat large molecules in the former case.^53^ However, calculations of the vertex correction using a density-fitting approach are considerably more expensive since indices, *i* and *a*, are on distinct integral factors.

Current code for the vertex corrected self-energies in eq 22 is not paralleized and slower than the code for the other self-energies. Hence we present vertex corrected IE for a subset of those given for the other methods in Tables 2 and S1 to S6.

**Table 2:** Ionization Energies and QP Weights for *t*-Butadiene, Furan, Pyrrole, and Thiophene Compared to Vertical Ionization Energies and ADC(3) Calculations^57,60^^,^^a^

orbital	symmetry	expt	HF	RPA	*Z*_RPA_	TDHF	*Z*_TDHF_	scTDHF	*Z*_scTDHF_	ADC(3)	*Z*_ADC(3)_
*t*-butadiene	*C*_2*h*_										
π_1_	*b*_*g*_	9.07	8.80	9.30	0.93	9.20	0.84	9.24	0.86	8.88	0.88
π_2_	*a*_*u*_	11.48	12.11	12.05	0.91	11.29	0.72	11.44	0.76	11.29/12.98	0.63/0.27
	*a*_*g*_	12.2	13.52	13.17	0.93	12.28	0.82	12.45	0.84	12.18	0.89
	*b*_*u*_	13.49	14.87	14.42	0.92	13.41	0.78	13.60	0.82	13.42	0.86
	*a*_*g*_	13.9	15.19	14.54	0.92	13.49	0.80	13.63	0.74	13.80	0.88
	*a*_*g*_	15.3	17.34	16.52	0.91	15.04	0.64	15.34	0.74	15.33	0.79
	*b*_*u*_	15.8	17.57	16.70	0.91	15.30	0.09	15.57	0.72	15.89/17.42	0.28/0.54
furan	*C*_2*v*_										
π_1_	*a*_1_	8.83	8.73	9.20	0.93	9.03	0.84	9.08	0.86	8.85	0.89
π_2_	*b*_1_	10.39	10.87	10.71	0.92	9.97	0.81	10.12	0.84	10.31	0.88
	*a*_1_	12.96	14.73	13.96	0.92	12.80	0.80	12.99	0.83	13.38	0.84
*n*_O_	*a*_1_	13.86	15.41	14.44	0.92	13.11	0.80	13.34	0.83	14.00	0.84
	*b*_2_	14.51	15.73	15.10	0.92	14.08	0.81	14.26	0.84	14.35	0.89
	*b*_2_	15.26	16.60	15.95	0.92	14.85	0.79	15.05	0.82	15.32	0.88
π_3_	*b*_1_	15.26	17.24	15.64	0.87	13.31/15.32	0.04	13.76	0.19	13.23/15.64	0.09/0.69
pyrrole	*C*_2*v*_										
π_1_	*a*_2_	8.02	8.12	8.53	0.93	8.28	0.84	8.35	0.86	8.05	0.89
π_2_	*b*_1_	9.05	9.44	9.44	0.92	8.78	0.82	8.92	0.84	8.95	0.88
	*a*_1_	12.85	14.36	13.73	0.92	12.67	0.81	12.85	0.83	13.11	0.90
π_3_	*b*_1_	12.85	15.47	14.28	0.92	12.27	0.17	12.65	0.03	12.73/14.26	0.30/0.43
	*b*_2_	13.57	14.92	14.27	0.87	13.25	0.80	13.57	0.75	13.52	0.89
	*a*_1_	14.27	16.13	15.37	0.92	14.21	0.08	14.43	0.64	14.82	0.89
	*b*_2_	14.80	15.88	15.23	0.92	14.18	0.77	14.33	0.82	14.53	0.88
thiophene	*C*_2*v*_										
π_1_	*a*_2_	8.85	8.93	9.26	0.92	8.96	0.83	9.04	0.85	8.84	0.88
π_2_	*b*_1_	9.49	9.44	9.55	0.93	9.10	0.83	9.21	0.85	9.06	0.89
*n*_*S*_	*a*_1_	12.00	12.93	12.50	0.92	11.67	0.81	11.82	0.83	11.91	0.88
π_3_	*b*_1_	12.46	14.23	13.43	0.89	11.88	0.30	12.16	0.57	12.52/13.83	0.57/0.15
	*b*_2_	13.11	14.36	13.91	0.92	13.03	0.81	13.20	0.84	13.35	0.89
	*a*_1_	13.80	15.04	14.31	0.91	13.16	0.79	13.35	0.82	13.60	0.89
	*b*_2_	14.23	15.70	14.96	0.91	13.77	0.65	13.98	0.81	14.20	
pyridine	*C*_2*v*_										
*n*_*N*_	*a*_1_	9.67	11.41	10.47	0.91	9.11	0.83	9.34	0.81	9.78	
π_1_	*a*_2_	9.85	9.48	9.91	0.93	9.64	0.81	9.72	0.85	9.44	
π_2_	*b*_1_	10.52	10.47	10.67	0.92	10.17	0.78	10.29	0.84	10.15	
	*b*_2_	12.60	14.17	13.62	0.92	12.63	0.82	12.80	0.84	12.73	
π_3_	*b*_1_	13.19	14.76	13.96	0.88	12.35	0.43	12.62	0.59	13.18	
	*a*_1_	13.79	15.71	14.79	0.91	13.38	0.70	13.71	0.78	13.92	
	*b*_2_	14.51	16.30	15.52	0.91	14.27	0.71	14.56	0.78	14.65	
	*a*_1_	15.6	17.82	16.88	0.90	15.47	0.05	15.74	0.23	16.02	
	*b*_2_	15.8	18.01	16.78	0.90	15.12	0.50	15.43	0.28	16.18	

a Experimental IE data are derived from refs (57), (65), and (66). QP weights are calculated at the HF eigenvalue energy. For small QP weights, the QP equation is solved numerically. All energies are in eV.

### Molecular Geometries and Basis Sets

The Quest database^31^ contains high-level quantum chemical calculations of excited state energies for seven categories of molecules. Quest-3 is the medium sized molecule category and it contains 27 molecules with four to six non-H atoms (C, N, O and S). For the present work, 21 of these molecules for which IE from photoemission are available in the literature were chosen. Here we use the molecular geometries given in the SI for ref. (31) which were determined at the CC3/aVTZ level. All calculations reported here were performed using the augmented cc-pVTZ (aVTZ) basis sets^54,55^ for each atom and corresponding auxiliary basis sets.^56^

In order to estimate the degree of convergence of IE values with basis set, IE calculations for *t*-butadiene, benzene and *s*-triazine were also performed using the augmented cc-VQZ (aVQZ) basis sets^54,55^ and auxiliary basis sets.^56^ IE for these systems typically increase by 0.1–0.2 eV going to the improved basis sets and are compared in SI Table S7.

## Results

### Ionization Energies

Vertical IE from photoemission data are compared to calculations of IE from HF eigenvalues (via Koopman’s theorem) and from *GW* self-energies in eq 20 to 22. Vertical IE from photoemission data correspond to binding energies at peak maxima.^57^ When vibrational excitations of the ionized state can be resolved, an adiabatic IE is the peak maximum of the 0–0 vibrational transition. For example, the first IE of *t*-butadiene is the *b*_*g*_ π_1_ state which has an adiabatic IE of 9.07 eV and a vertical IE of 9.29 eV.^57^ The former is judged to be the 0–0 line in a well-resolved He I spectrum and the latter the maximum in a broad peak using 80 eV synchrotron radiation.

Numerical values of IE from experiment and the various theoretical methods used are presented in Table 2 for *t*-butadiene, furan, pyrrole, thiophene and pyridine as there are combined experimental and ADC(3) theoretical studies in the literature for these systems. IE values and QP weights (or pole strengths) from ADC(3) and the *GW* methods used here are compared for these molecules. ADC(3) has been benchmarked against families of diagonal^58^ and nondiagonal^59^ self-energy methods. Compared to IP-EOM-CCSDT in a cc-pVTZ basis,^58^ it was found to have a MAE of 0.32 eV for a database of 80 IE.

Values of IE from all systems studied here are given in Supporting Information Tables S1 to S6. In the main text we present differences in experimental vertical IE and IE from HF eigenvalues and *GW* calculations (ΔIE) in graphical and tabular formats for nonbonding N and O lone pair (*n*_*N*_ and *n*_*O*_) frontier states and all occupied π states. States are numbered from lowest to highest binding energy as π_1_, π_2_, etc. A positive value for ΔIE corresponds to an overestimate of the experimental IE.

Accuracies of estimates of experimental IE of various types of frontier orbital state derived from HF eigenvalues depend markedly on the orbital character of the state. The first π state IE is typically underestimated by a few tenths of an eV while the second π IE is overestimated by slightly more (Tables 3,5, and 7). HF overestimates π_3_ levels in six membered rings by up to 2.6 eV (Table 7). These large ΔIE^*HF*^ values imply a need for large self-energy corrections and inherently larger errors in IE after *GW* self-energy corrections. Deeper π levels are typically relatively unimportant configurations in low lying excited states of molecules. Consequently these errors are relatively unimportant in predicting their visible and UV spectra. However, as noted in the Introduction, ΔIE^*HF*^ values for *n*_*N*_ and *n*_*O*_ nonbonding levels are large. These are frontier orbitals and are therefore important in predicting optical spectra of the molecules, especially *n* to π* transitions.

**Table 3:** ΔIE for π States in Alkenes and Carbonyl Molecules in eV

orbital	symmetry	ΔIE^HF^	ΔIE^RPA^	ΔIE^TDHF^	ΔIE^scTDHF^	ΔIE^vTDHF^	ΔIE^scvTDHF^
butadiene	*C*_2*v*_						
π_1_	*b*_*g*_	–0.27	0.23	0.13	0.17	–0.28	–0.22
π_2_	*a*_*u*_	0.63	0.57	–0.19	–0.04	–0.03	0.10
isobutene	*C*_2*v*_						
π_1_	*b*_1_	0.01	0.27	0.01	0.08	–0.29	–0.21
MCP	*C*_2*v*_						
π_1_	*b*_1_	–0.33	0.10	–0.10	–0.04	–0.42	–0.36
acetone	*C*_2*v*_						
π_CO_	*b*_1_	0.72	0.39	–0.47	–0.27	–0.42	–0.27
acrolein	C_*s*_						
π_CC_	*a*″	0.02	0.37	0.10	0.18	–0.15	–0.07
π_CO_	*a*″	1.1	0.7	–0.4	–0.2	–0.1	0.1
glyoxal	C_2*h*_						
π_CO_		0.5	0.4	–0.3	–0.1	–0.3	–0.1
π_CO_		0.8	0.6	–0.1	–0.0	0.0	0.1
MSE π_1_, π_2_, π_CC_		0.01	0.31	–0.01	0.07	–0.23	–0.15
MAE π_1_, π_2_, π_CC_		0.25	0.31	0.11	0.10	0.23	0.19
MSE π_CO_		0.78	0.53	–0.33	–0.14	–0.20	–0.04
MAE π_CO_		0.78	0.53	0.33	0.14	0.20	0.14

**Table 4:** ΔIE for Nonbonding Oxygen and Sulfur States in eV

orbital	symmetry	ΔIE^HF^	ΔIE^RPA^	ΔIE^TDHF^	ΔIE^scTDHF^	ΔIE^vTDHF^	ΔIE^scvTDHF^
acetone	*C*_2*v*_						
*n*_1_	*b*_2_	1.60	0.68	–0.71	–0.47	–0.57	–0.36
acrolein	*C*_*s*_						
*n*_1_	*a*′	1.69	0.71	–0.74	–0.49	–0.59	–0.37
glyoxal	*C*_2*h*_						
*n*_1_	*a*_*g*_	1.5	0.8	–0.5	–0.3	–0.5	–0.3
*n*_2_	*b*_*u*_	2.16	0.87	–0.84	–0.57	–0.47	–0.23
furan	*C*_2*v*_						
*n*_1_	*a*_1_	1.55	0.58	–0.75	–0.52	–0.69	–0.46
MSE *n*_1_, *n*_2_		1.70	0.55	–0.71	–0.47	–0.55	–0.34
MAE *n*_1_, *n*_2_		1.70	0.55	0.71	0.47	0.55	0.34
thiophene	*C*_2*v*_						
*n*_1_	*a*_1_	0.93	0.50	–0.33	–0.18	–0.37	–0.24

**Table 5:** ΔIE for π Bond Pairs and *n*_N_ Lone Pairs in Diacetylene, Cyanoacetylene, and Cyanogen in eV

orbital	symmetry	ΔIE^HF^	ΔIE^RPA^	ΔIE^TDHF^	ΔIE^scTDHF^	ΔIE^vTDHF^	ΔIE^scvTDHF^
diacetylene	*D*_*∞h*_						
π_1_	π_*g*_	–0.10	0.30	0.20	0.24	–0.19	–0.14
π_2_	π_*u*_	0.78	0.57	–0.20	–0.08	–0.09	0.02
cyanoacetylene	*C*_*∞v*_						
π_1_	π	0.07	0.43	0.30	0.35	–0.05	0.00
π_2_	π	0.86	0.68	–0.06	0.06	0.05	0.15
*n*_1_	σ	2.58	1.09	–0.78	–0.51	–0.31	–0.09
cyanogen	*D*_*∞h*_						
π_1_	π_*g*_	0.14	0.46	0.30	0.36	–0.02	0.04
π_2_	π_*u*_	0.86	0.76	0.12	0.23	0.20	0.29
*n*_1_	σ_*g*_	2.52	1.13	–0.69	–0.44	–0.23	–0.03
*n*_2_	σ_*u*_	2.58	1.12	–0.76	–0.50	–0.25	–0.05
MSE π_1_		0.04	0.40	0.26	0.32	–0.09	–0.03
MAE π_1_		0.10	0.40	0.26	0.32	0.09	0.06
MSE π_2_		0.83	0.67	–0.05	0.07	0.05	0.15
MAE π_2_		0.83	0.67	0.13	0.12	0.11	0.15
MSE *n*_1,2_		2.56	1.11	–0.74	–0.48	–0.26	–0.06
MAE *n*_1,2_		2.56	1.11	0.74	0.48	0.26	0.06

**Table 6:** ΔIE for Nitrogen Lone Pairs in Five and Six Membered Rings in eV

orbital	symmetry	ΔIE^HF^	ΔIE^RPA^	ΔIE^TDHF^	ΔIE^scTDHF^	ΔIE^vTDHF^	ΔIE^scvTDHF^
imidazole	*C*_*s*_						
*n*_1_	*a*′	1.6	0.6	–0.9	–0.6	–0.6	–0.4
pyridine	*C*_2*v*_						
*n*_1_	*a*_1_	1.74	0.80	–0.56	–0.33	–0.47	–0.27
pyrazine	*D*_2*h*_						
*n*_1_	*a*_*g*_	1.68	0.86	–0.40	–0.18	–0.37	–0.18
*n*_2_	*b*_1*u*_	2.30	0.88	–0.80	–0.55	–0.49	–0.23
pyridazine	*C*_2*v*_						
*n*_1_	*b*_2_	1.84	0.79	–0.66	–0.42	–0.53	–0.30
*n*_2_	*a*_1_	1.79	0.90	–0.42	–0.20	–0.41	–0.18
pyrimidine	*C*_2*v*_						
*n*_1_	*b*_2_	1.68	0.77	–0.58	–0.34	–0.48	–0.28
*n*_2_	*a*_1_	1.72	0.73	–0.66	–0.42	–0.51	–0.31
*s*-triazine	*D*_3*h*_						
*n*_1_, *n*_2_	*e*′	1.64	0.73	–0.63	–0.38	–0.49	–0.27
*n*_3_	*a*_1_^′^	2.39	0.89	–0.79	–0.56	–0.40	–0.11
*s*-tetrazine	*D*_2*h*_						
*n*_1_	*b*_3*g*_	1.73	0.83	–0.48	–0.26	–0.44	–0.26
*n*_2_	*b*_1*u*_	2.55	1.13	–0.23	–0.27		
*n*_3_	*b*_2*u*_	2.63	1.25	–0.29	–0.16		
*n*_4_	*a*_*g*_	1.03	0.29	–0.90	–0.69		
MSE *n*_1–4_		1.88	0.82	–0.59	–0.38	–0.47	–0.25
MAE *n*_1–4_		1.88	0.82	0.59	0.38	0.47	0.25

**Table 7:** ΔIE for π Bond Pairs in Five and Six Membered Rings in eV

orbital	symmetry	ΔIE^HF^	ΔIE^RPA^	ΔIE^TDHF^	ΔIE^scTDHF^	ΔIE ^vTDHF^	ΔIE ^scvTDHF^
cyclopentadiene	*C*_2*v*_						
π_1_	*a*_2_	–0.22	0.23	0.09	0.13	–0.25	–0.19
π_2_	*b*_1_	0.53	0.55	–0.04	–0.08	–0.08	0.02
π_3_	*b*_1_	1.2	0.5	–1.5	–0.8		
furan	*C*_2*v*_						
π_1_	*a*_2_	–0.10	0.37	0.20	0.25	–0.05	0.01
π_2_	*b*_1_	0.48	0.32	–0.43	–0.27	–0.30	–0.17
π_3_	*b*_1_	1.98	0.38	–1.95	–1.50		
imidazole	*C*_*s*_						
π_1_	*a*″	–0.01	0.42	0.19	0.27	0.01	0.06
π_2_	*a*″	0.62	0.42	–0.43	–0.25	–0.23	–0.11
π_3_	*a*′	2.43	1.81	–0.75	0.94		
pyrrole	*C*_2*v*_						
π_1_	*a*_2_	0.10	0.51	0.26	0.33	0.09	0.15
π_2_	*b*_1_	0.39	0.39	–0.27	–0.13	–0.17	–0.07
π_3_	*b*_1_	2.62	1.43	–0.58	–0.20		
thiophene	*C*_2*v*_						
π_1_	*a*_2_	0.08	0.41	0.11	0.19	–0.05	0.01
π_2_	*b*_1_	–0.05	0.06	–0.39	–0.28	–0.38	–0.31
π_3_	*b*_1_	1.77	0.97	–0.58	–0.30		
benzene	*D*_6*h*_						
π_1,2_	*e*_1*g*_	–0.12	0.23	–0.11	–0.02	–0.15	–0.09
π_3_	*a*_2*u*_	1.55	0.84	–0.67	–0.25		
pyridine	*C*_2*v*_						
π_1_	*a*_2_	–0.37	0.06	–0.21	–0.13	–0.30	–0.24
π_2_	*b*_1_	–0.05	0.15	–0.35	–0.23	–0.32	–0.21
π_3_	*b*_1_	1.57	0.77	–0.84	–0.57		
pyrazine	*D*_2*h*_						
π_1_	*b*_1*g*_	–0.39	0.14	–0.03	0.04	–0.18	–0.13
π_2_	*b*_2*g*_	0.12	0.19	–0.44	–0.28	–0.30	–0.19
π_3_	*b*_3*u*_	1.77	0.93	–0.80	–0.50		
pyridazine	*C*_2*v*_						
π_1_	*a*_2_	–0.13	0.25	–0.07	0.03	–0.13	–0.06
π_2_	*b*_1_	–0.16	0.11	–0.33	–0.21	–0.30	–0.21
π_3_	*b*_1_	1.77	0.89	–0.81	–0.51		
pyrimidine	*C*_2*v*_						
π_1_	*b*_1_	–0.18	0.21	–0.09	0.00	–0.16	–0.10
π_2_	*a*_2_	0.14	0.21	–0.42	–0.26	–0.30	–0.20
π_3_	*b*_1_	1.84	1.01	–0.61	–0.34		
*s*-triazine	*D*_3*h*_						
π_1,2_	*e*″	0.22	0.34	–0.26	–0.09	–0.16	–0.05
π_3_	*a*_2_^′^	2.06	1.23	–0.09	0.06		
*s*-tetrazine	*D*_2*h*_						
π_1_	*b*_2*g*_	–0.18	0.32	0.09	0.18	–0.11	–0.05
π_2_	*b*_1*g*_	0.06	0.17	–0.43	–0.29	–0.29	–0.18
π_3_	*b*_3*u*_	1.80	0.91	–0.88	–0.58		
MSE π_1_		–0.11	0.29	0.02	0.09	–0.12	–0.05
MAE π_1_		0.16	0.29	0.15	0.14	0.14	0.10
MSE π_2_		0.18	0.28	–0.32	–0.20	–0.25	–0.16
MAE π_2_		0.26	0.28	0.32	0.20	0.25	0.15
MSE π_3_		1.86	0.97	–0.83	–0.37		
MAE π_3_		1.86	0.97	0.83	0.54		

Combined photoionization and ADC(3) studies of ionization in *t*-butadiene^57^ and furan, pyrrole and thiophene^60^ are available. ADC(3) and SAC–CI results are available for pyridine, an example of a six membered ring with a nonbonding N lone pair first IE. Comparison of ADC(3) results with the methods used here is made in Table 2. What emerges is that predictions of IE from Σ^*TDHF*^, Σ^*scTDHF*^ and ADC(3) are generally in agreement and that IE predictions from Σ^*scTDHF*^ agree much better with ADC(3) than Σ^*RPA*^ and ADC(3). In cases where ADC(3) predicts more than one significant QP weight for a particular ionization, owing to strong configuration interaction, there is usually a reduction in the QP weight, *Z*, for the corresponding ionization in Σ^*scTDHF*^, while there is little reduction in QP weight in Σ^*RPA*^. Reduction in QP weight occurs because of multiple roots in the QP equation for that state. Pole positions in Σ^*scTDHF*^ self-energies are several eV closer to QP energies than those in Σ^*RPA*^. Hence shakeup excitations are more likely in Σ^*scTDHF*^, compared to Σ^*RPA*^ self-energies. In each of the four molecules considered, the lowest occupied π state shows strong configuration interaction and at least one moderately strong pole in ADC(3).

### Butadiene, Acrolein, Glyoxal, and Acetone

**Figure 2:**
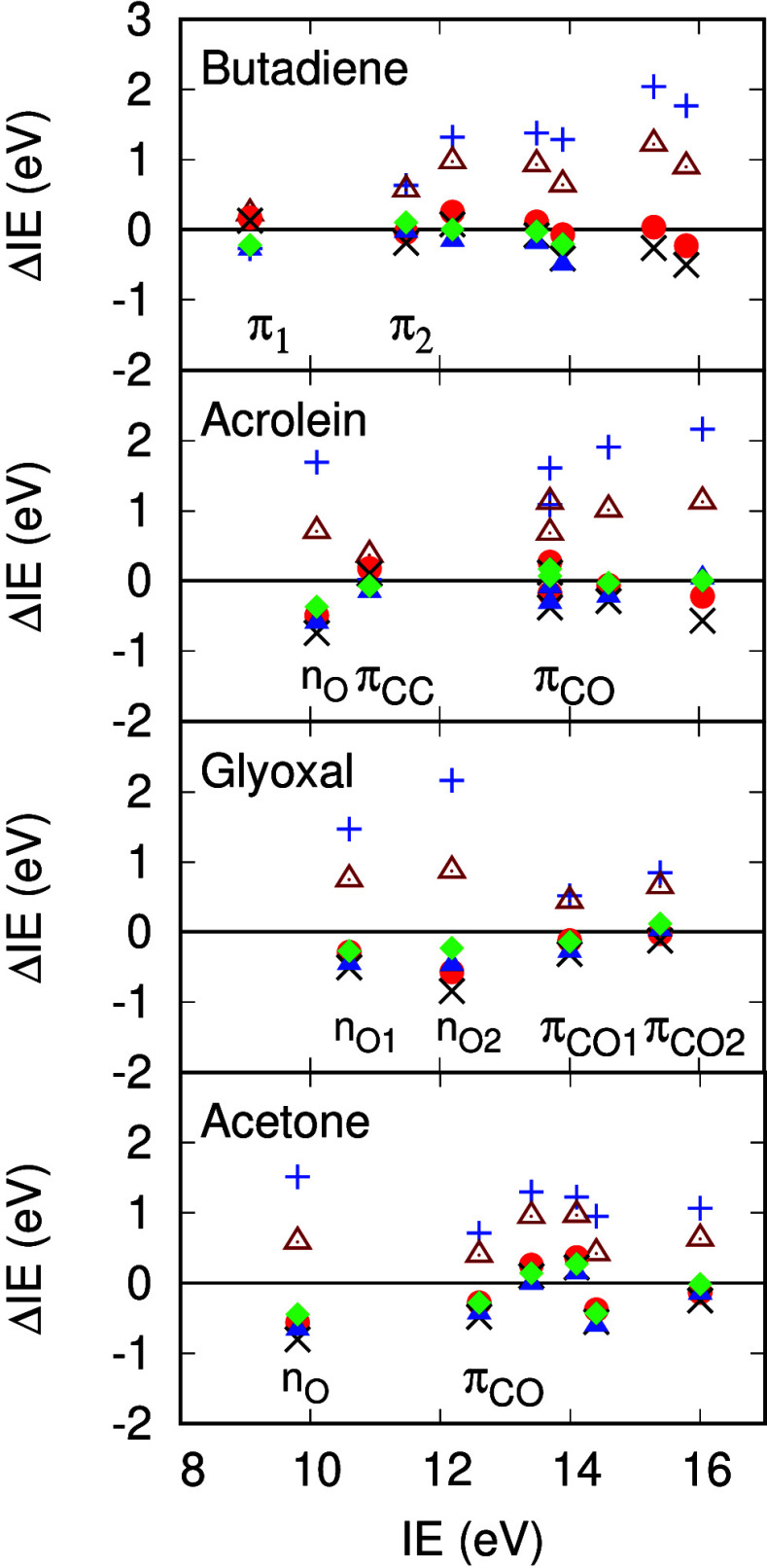
ΔIE for *t*-butadiene, acrolein, glyoxal, and acetone in eV. ΔIE^HF^ (blue +), ΔIE^RPA^ (brown triangles), ΔIE^TDHF^ (black X), ΔIE^scTDHF^ (red circles), ΔIE^vTDHF^ (blue triangles), ΔIE^scvTDHF^ (green diamonds). Orbital character is indicated as π bonding or nonbonding O lone pairs. Experimental IE data are derived from refs (57), (62−64).

The first IE of *t*-butadiene is the *b*_*g*_ π_1_ state with an adiabatic IE of 9.07 eV and a vertical IE of 9.29 eV.^57^ HF underestimates the vertical IE by 0.49 eV (Tables 2 and 3), while the self-energy corrected methods are in good agreement with the vertical IE value, ranging from 9.18 to 9.30 eV (Table 2 and Figure 2).

ADC(3) results for *t*-butadiene by Holland and co-workers^57^ placed the π_1_ state at 8.88 eV and were confirmed by Deleuze and Knippenberg^61^ who reported an ADC(3) value of 8.98 eV in a larger basis set. The vertical IE (maximum intensity in photoemission peak) of 9.29 eV for the IE of the highest occupied π_1_ state results in a ΔIE^*RPA*^ value of 0.01 eV while using the adiabatic value of 9.07 eV (0–0 line in photoemission) in this case results in a ΔIE^*RPA*^ value of 0.23 eV, which is in better agreement with the ΔIE^*RPA*^ MSE value of 0.26 eV for π_1_ states in Table 3. Hence, we use the adiabatic value of 9.07 eV as the first IE for *t*-butadiene.

**Figure 3:**
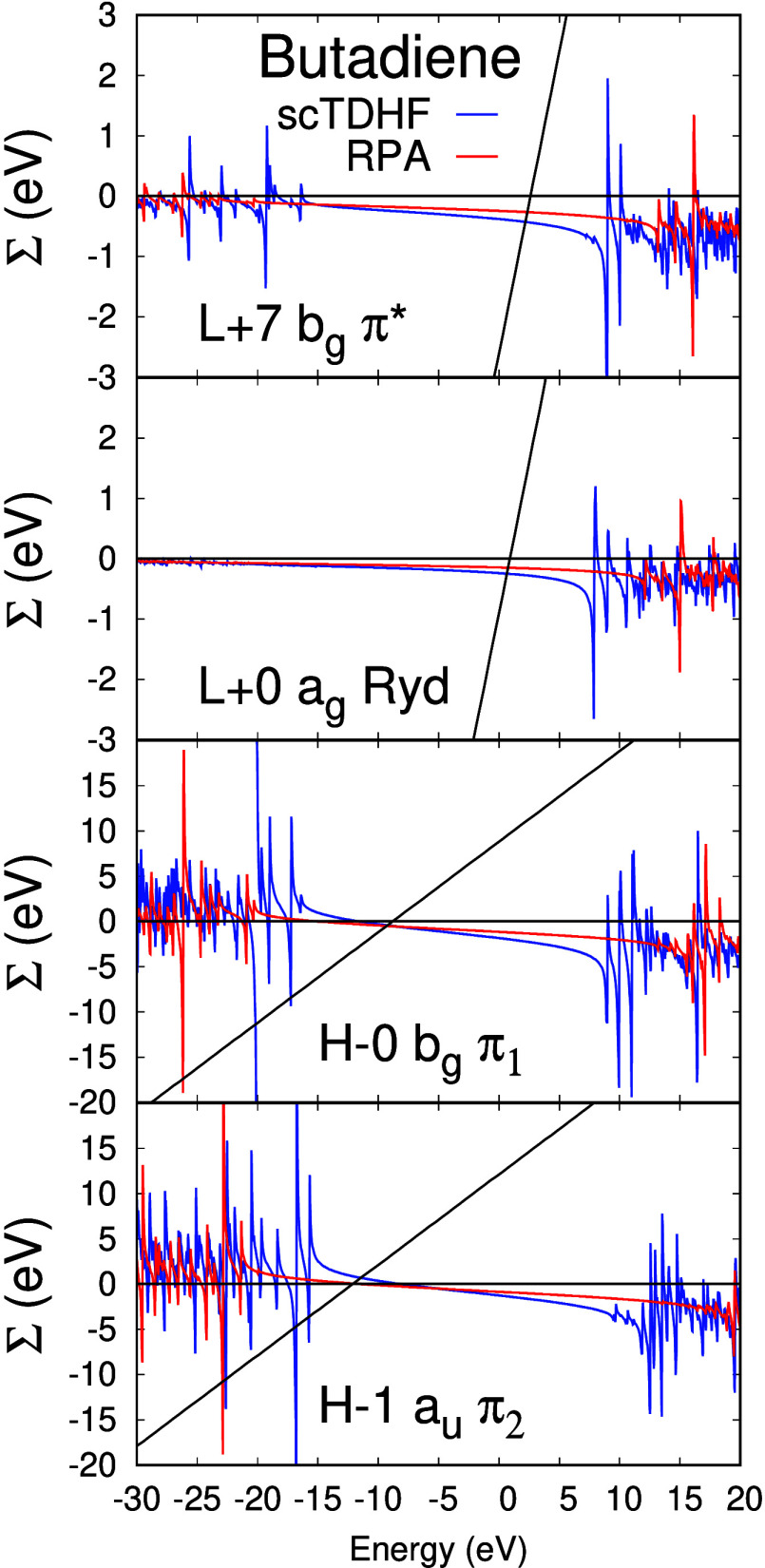
Self-energies as a function of energy for *t*-butadiene for the π_1_ and π_2_*H* – 0 and *H* – 1 occupied states, the *L* + 0 state of Rydberg character and the lowest state of π* character, *L* + 7. Blue (red) curves are self-energies from scTDHF (RPA) calculations. Intersections of solid black lines with self-energy curves indicate solutions to the QP equation (eq 23).

Energy dependences of the RPA and scTDHF self-energies for the two occupied π states, *L* + 0 state and the lowest π* state in *t*-butadiene are shown in Figure 3. The main difference in the plots is that pole positions are shifted toward the center of the plot in Σ^*scTDHF*^ compared to Σ^*RPA*^ by over 5 eV. Polarizability pole positions, Ω_+_^α^, which appear in denominators of the self-energies in eq 20 to 22 occur at lower energies in Σ^*scTDHF*^ owing to electron–hole attraction in the polarizability calculation. Self-energies at the QP energy for the π_1_*H* – 0 state are almost the same (around −0.5 eV) for both methods. Self-energies for the π_2_, *H* – 1 state in Figure 3 differ considerably. There is a pole in Σ^*scTDHF*^ with a large residue around 5 eV below the π_2_ QP energy. This contributes to the reduced QP weight of 0.76 for this state from Σ^*scTDHF*^ (Table 2). This compares to QP weights of 0.63 and 0.27 for IE at 11.29 and 12.98 in ADC(3). The Σ^*scTDHF*^ π_2_ IE is 11.44 eV, in good agreement with experiment (11.48 eV, SI Table S1) and the ADC(3) IE at 11.29 eV. In contrast, Σ^*RPA*^ predicts an IE of 12.05 eV and a QP weight of 0.91. Σ^*TDHF*^ predicts an IE of 11.29 eV and a QP weight of 0.72, similar to values from scTDHF and ADC(3).

Inspection of the next four IE of *t*-butadiene shows that there are no significant shakeup contributions and QP weights from TDHF, scTDHF and ADC(3) are in good agreement with QP weights around 0.7 to 0.8; RPA QP weights remain above 0.9 for these states. Differences in experimental, ADC(3), TDHF and scTDHF IE are all below 0.3 eV, while RPA overestimates these IE by 0.6 to 1.2 eV (Figure 2). The next IE with a significant shakeup weight is the *b*_*u*_ IE found at 15.8 eV in experiment (Table 2). ADC(3) predicts QP weights of 0.28 and 0.54 for IE at 15.89 and 17.42 eV for hole states with this symmetry. This compares to a QP weight of 0.72 in scTDHF and an IE of 15.57 eV, in good agreement with the experimental value. RPA overestimates the binding energy for this IE by 0.9 eV. For TDHF the QP weight collapses to 0.09 at the HF eigenvalue energy because of proximity to a self-energy pole. ΔIE^*vTDHF*^ and ΔIE^*scvTDHF*^ for *t*-butadiene in Table 3 are larger than for ΔIE^*TDHF*^ and ΔIE^*scTDHF*^, indicating that vertex corrections shift the IE toward the experimental adiabatic IE of 9.07 eV. The vertex corrected IE values, 8.79 and 8.85 eV, are in reasonable agreement with the ADC(3) values in refs. (57) and (61) 8.88 and 8.98 eV, and the adiabatic photoemission IE of 9.07 eV.

Replacing one or two terminal CH_2_ fragments in *t*-butadiene by O yields acrolein and glyoxal with π_*CO*_ bonds replacing π_*CC*_ bonds. ΔIE values for acrolein and glyoxal are given in Tables 3 and 4. These are illustrated graphically in Figure 2. The first IE in experiment in acrolein is the *a*′ *n*_*O*_ state at 10.10 eV and the second IE is the π_*CC*_ state at 10.92 eV (SI Table S1). HF eigenvalues are in reverse of this order. The Koopmans HF eigenvalue prediction of 10.94 eV for the π_*CC*_ level is in good agreement with experiment, while the *n*_*O*_ state binding energy is overestimated by 1.69 eV (Table 4). Adding the RPA self-energy to the HF eigenvalue reduces this overestimate to 0.70 eV; the TDHF(scTDHF) self-energy overcorrects by 0.74(0.49) eV. The RPA self-energy overestimates the binding energy for the π_*CC*_ state in acrolein by 0.37 eV. The TDHF self-energy overestimates it by 0.10 eV and the scTDHF self-energy underestimates it by 0.18 eV (Table 3).

There are similar patterns in ΔIE values in glyoxal and acetone: the two π_*CO*_ IE in glyoxal are overestimated by the RPA self-energy by 0.4 and 0.6 eV, while the scTDHF self-energy underestimates them by 0.12 and 0.02 eV (Table 3). Similarly, the π_*CO*_ IE in acetone is overestimated by Σ^*RPA*^ by 0.4 eV, while the Σ^*scTDHF*^ underestimates it by 0.3 eV. Tables 3 and 4 show that the minimum MSE and MAE for π states in the smaller molecules in this study are found for Σ^*scTDHF*^; for nonbonding O states the minimum MSE and MAE are found for Σ^*scvTDHF*^.

### Double and Triple Bonded Molecules

**Figure 4:**
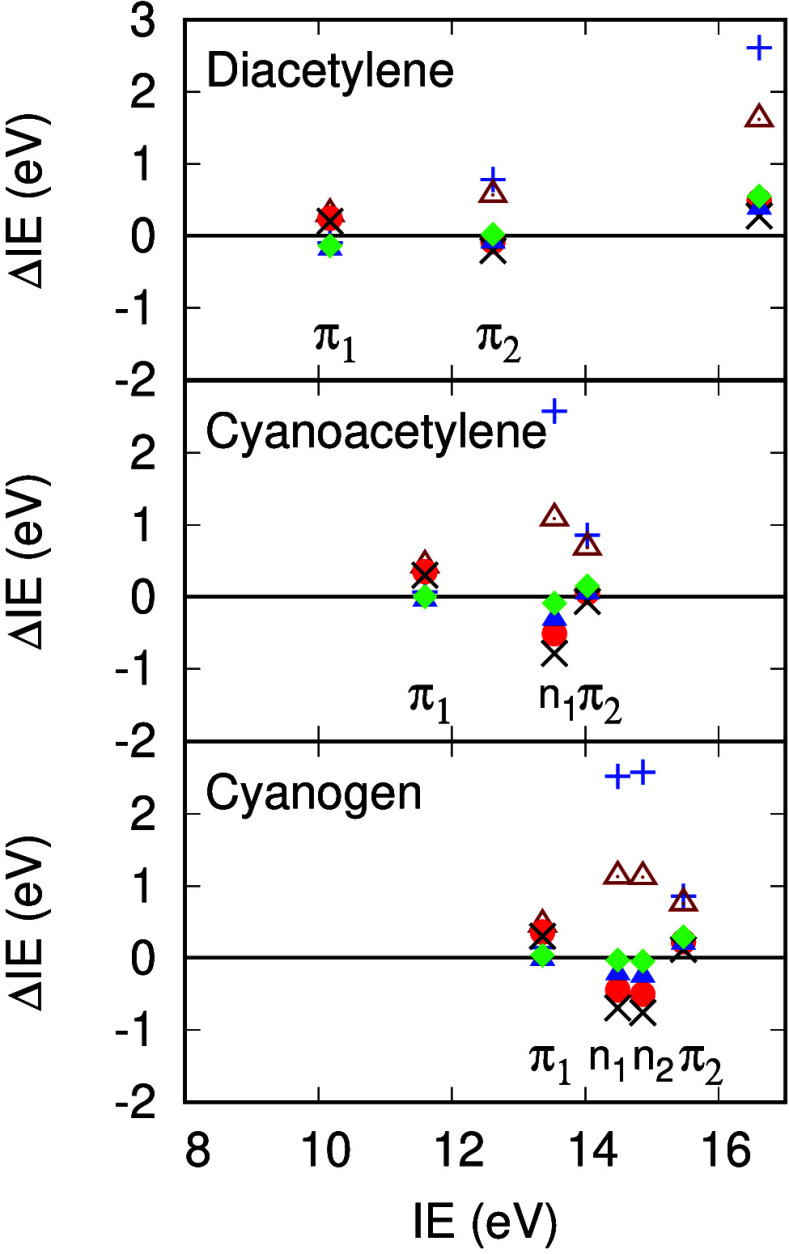
ΔIE for diacetylene, cyanoacetylene, and cyanogen in eV. ΔIE^HF^ (blue +), ΔIE^RPA^ (brown triangles), ΔIE^TDHF^ (black X), ΔIE^scTDHF^ (red circles), ΔIE^vTDHF^ (blue triangles), ΔIE^scvTDHF^ (green diamonds). Symmetry labels indicate the orbital character as π bonding or N nonbonding lone pairs. Experimental IE data are derived from ref (67).

**Figure 5:**
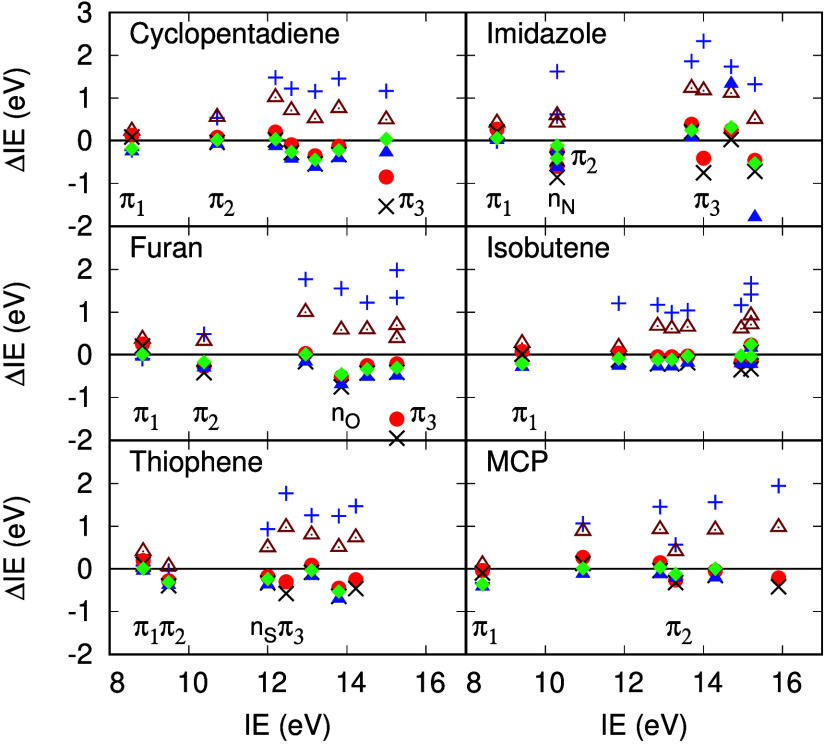
ΔIE for five membered rings, isobutene, and methylene cyclopropene (MCP) in eV. ΔIE^HF^ (blue +), ΔIE^RPA^ (brown triangles), ΔIE^TDHF^ (black X), ΔIE^scTDHF^ (red circles), ΔIE^vTDHF^ (blue triangles), and ΔIE^scvTDHF^ (green diamonds). Experimental IE data are derived from refs (65), (68−71).

Figure 4 shows differences in calculated and experimental IE values for the isoelectronic series: diacetylene, cyanoacetylene and cyanogen and Figure 5 shows similar data for isobutene and methylenecyclopropene (MCP). The triply bonded acetylene series shows that relaxation and correlation effects in IE in π and σ bonds and *n*_*N*_ lone pairs differ markedly, as was the case for *n*_*O*_ states in acrolein and glyoxal. This can partly be attributed to the high degree of localization of lone pair states.

ΔIE^*HF*^ for π_1_ states in the acetylenes are small (Table 5); addition of the RPA self-energy results in larger ΔIE^*RPA*^ values, up to 0.5 eV; addition of screened electron–hole exchange in the polarizability and vertex corrections to the self-energy reduces ΔIE values close to zero again. ΔIE^*HF*^ for π_2_ states are of order 0.8 eV in the acetylenes and self-energy corrections reduce these differences.

Table 5 also shows ΔIE for *n*_*N*_ lone pair states in cyanoacetylene and cyanogen. ΔIE^*HF*^ values are around 2.5 eV, ΔIE^*RPA*^ values reduce to around 1.1 eV and ΔIE^*scTDHF*^ overshoot by around 0.5 eV. Addition of vertex corrections to the self-energies reduces ΔIE values, as can be seen graphically in Figure 4. *n*_*N*_ states in five and six membered rings also have large ΔIE^*HF*^ and there are similar patterns in ΔIE^*RPA*^ and ΔIE^*scTDHF*^ to those found in cyanoacetylene and cyanogen. Vertex corrections for π_1_ states reduce the IE by 0.3 to 0.4 eV in *t*-butadiene, isobutene, MCP, diacetylene, cyanoacetylene and cyanogen.

**Figure 6:**
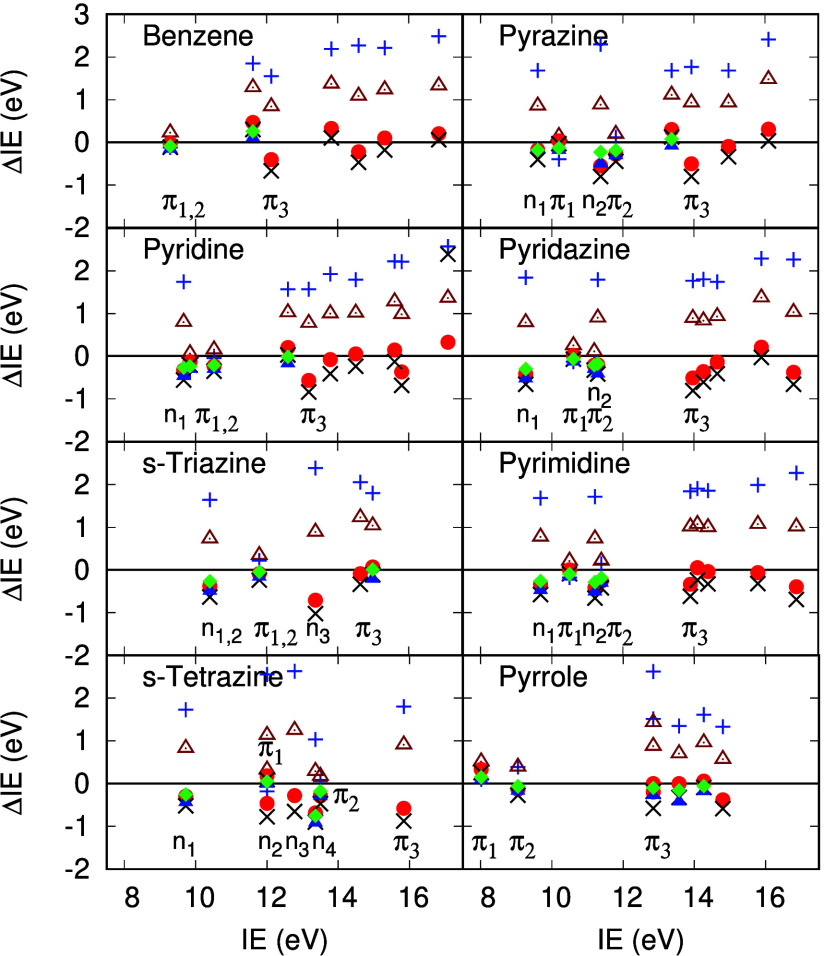
ΔIE for six membered rings in eV. HF Koopmans value (blue +), ΔIE^RPA^ (brown triangles), ΔIE^TDHF^ (black X), ΔIE^scTDHF^ (red circles), ΔIE^vTDHF^ (blue triangles), ΔIE^scvTDHF^ (green diamonds). Experimental IE data are derived from refs (65), (66), (72), and (74).

ΔIE^*HF*^ for the π_1_ states in isobutene and MCP are small. Figure 5 and Table 3 show that there is excellent agreement between scTDHF and experiment for nearly all IE to 16 eV for these alkenes. This is so even though ΔIE^*HF*^ and the required self-energy correction are as large as 1.5 eV. The absence of nonbonded electron pairs in *t*-butadiene, (Figure 2), diacetylene (Figure 4), cyclopentadiene, isobutene and MCP (Figure 5) and benzene (Figure 6) seems to correlate with improved IE predictions by Σ^*scTDHF*^ (Tables 3, 5 and 7).

### Five Membered Rings

Experimental and calculated IE and QP weights from the methods used here and ADC(3)^60^ for furan, pyrrole and thiophene are given in Table 2. ΔIE for five membered rings are illustrated in Figure 5 (cyclopentadiene, furan, thiophene and imidazole) and Figure 6 (pyrrole) and numerical values are given in Tables 4, 6 and 7. ΔIE^*HF*^ for cyclopentadiene, furan, thiophene, pyrrole and imidazole π states are qualitatively similar: ΔIE^*HF*^ for the π_1_ state is small, it increases to around 0.5 eV for π_2_ (with the exception of thiophene) and ranges from 1.2 to 2.6 eV for the π_3_ state. In the heterocycles the π_3_ state is localized on the heteroatom.

Agreement between theory and experiment is generally good for the five membered rings, except for π_3_ levels. Inspection of the columns labeled ADC(3) and *Z*_*ADC(3)*_ in Table 2 shows that QP weights are typically around 0.9 for states with IE up to 15 eV, except for π_3_ states, where there are at least two QP with significant weight, indicating a breakdown of the single particle picture of ionization and existence of shakeup excitations. Inspection of the *Z*_*RPA*_ column shows that QP weights in Σ^*RPA*^ calculations remain around 0.9 for all IE up to around 15 eV. In contrast, QP weights from Σ^*scTDHF*^ calculations show a marked reduction in *Z*_*scTDHF*_ for states where ADC(3) predicts shakeup excitations. In furan the *b*_1_π_3_*Z*_*scTDHF*_ weight falls to 0.19. Similar breakdowns of the single particle picture of ionization also occur in the π_3_ states of pyrrole and thiophene, where *Z*_*scTDHF*_ QP weights fall to 0.03 and 0.57, respectively. Corresponding values for *Z*_*RPA*_ are 0.87, 0.92 and 0.89, so that the single particle picture breakdown is not captured by Σ^*RPA*^.

### Six Membered Rings

Azine derivatives of benzene present a challenge to perturbative methods for IE since because of the strong relaxation and correlation effects associated with nonbonding N lone pair states,^18^ as mentioned above. In each of the six azines studied here (pyridine, pyrazine, pyridazine, pyrimidine, *s*-triazine and *s*-tetrazine) the first IE is always the nonbonding *n*_1_ state. HF eigenvalue estimates of the first IE of these azines exceed vertical IE values by between 1.64 and 1.84 eV. In contrast the π_1_ level is mostly underestimated by HF eigenvalues, by up to 0.39 eV in pyrazine, and overestimated by 0.22 eV in *s*-triazine. The order of experimental vertical IE in azines assigned in ref. (66) and ref. (72) is given in Table 8. HF Koopmans values for nonbonding levels in these azines are shifted to higher binding energy relative to experimental vertical IE, so that the HF π_1_ level is the *H* – 0 level, with the exception of *s*-tetrazine. After a Σ^*RPA*^ self-energy correction, the order of the *n*_1_ and π_1_ levels remains reversed compared to the experimental assignment in pyridine, pyrazine and pyrimidine. After a Σ^*scTDHF*^ self-energy correction, the order of all nonbonding and π levels is in agreement with the experimental vertical IE order given in Table 8. ΔIE^*RPA*^ values for *n*_*N*_ states in the azines range from 0.73 eV in pyrimidine and *s*-triazine to 1.25 eV in *s*-tetrazine, with MSE and MAE both 0.82 eV. ΔIE^*scTDHF*^ values range from −0.18 eV in pyrazine to −0.69 in *s*-tetrazine with MSE and MAE of −0.38 and 0.38 eV (Table 6). Addition of the vertex correction term to either TDHF or scTDHF reduces the magnitude the MAE to 0.38 and 0.25 eV, respectively.

**Table 8:** Vertical IE of Six Membered Rings in Order of Binding Energy As Assigned in Refs (66) and (72)

molecule	vertical IE level order and character
pyridine	*a*_1_*n*_1_ > *a*_2_π_1_ > *b*_1_π_2_ > *b*_1_π_3_
pyrazine	*a*_*g*_*n*_1_ > *b*_1*g*_π_1_ > *b*_1*u*_*n*_2_ > *b*_2*g*_π_2_ > *b*_3*u*_π_3_
pyridazine	*b*_2_*n*_1_ > *a*_2_π_1_ > *b*_1_π_2_ > *a*_1_*n*_2_ > *b*_1_π_3_
pyrimidine	*b*_2_*n*_1_ > *b*_1_π_1_ > *a*_1_*n*_2_ > *a*_2_π_2_ > *b*_1_π_3_
*s*-triazine	*e*′*n*_1_ > *e*″π_1,2_ > *a*_1_^′^*n*_2_ > *a*_2_^′^π_3_
*s*-tetrazine	*b*_3*g*_*n*_1_ > *b*_2*g*_π_1_ > *b*_1*u*_*n*_2_ > *a*_*g*_*n*_3_ > *b*_2*u*_*n*_4_ > *b*_1*g*_π_2_ > *b*_3*u*_π_3_

In pyridine, ADC(3)^73^ predicts the highest nonbonding and π levels in the order *a*_2_π_1_ > *a*_1_*n*_*N*_ > *b*_1_π_2_, while SAC–CI agrees with scTDHF and the experimental order, *a*_1_*n*_*N*_ > *a*_2_π_1_ > *b*_1_π_2_. The difference in experimental IE values for these states is less than 0.2 eV (Table 2). ADC(3) QP weights were not reported in ref. (73)

There is generally good agreement between π_1_ and π_2_ levels from Σ^*RPA*^, Σ^*scTDHF*^ and experimental vertical IE for the azines (Table 7). Σ^*RPA*^ tends to overestimate binding energies by up to 0.3 eV while Σ^*scTDHF*^ underestimates them by similar amounts. The π_3_ level in the azines shows a significantly reduced QP weight, *Z*_*scTDHF*_ (Table 2), as in the five membered ring heterocycles considered above. Values are pyridine (0.58), pyrazine (0.29), pyridazine (0.60), pyrimidine (0.63), *s*-triazine (0.68) and *s*-tetrazine (0.56). ADC(3) predicts a QP weight of 0.61 in *s*-tetrazine.^72^ The π_3_ level binding energy is overestimated by Σ^*RPA*^ by between 0.8 eV in benzene and 1.2 eV in *s*-triazine, while the difference in binding energy is significantly less than this in Σ^*scTDHF*^ (Table 7).

We note here that π_3_ states exhibit strong shakeup effects which complicate the analysis of these binding energies. There is good agreement between ADC(3) and scTDHF for pyridine IE (Table 2), except where the scTDHF QP weight falls below 0.5 and the single particle picture fails. Larger ΔIE values for π_3_ levels in five and six membered rings are clearly evident in Figure 5 and 6, especially in furan, imidazole, benzene, pyridine, pyrazine and pyridazine.

### Effects of Electron–Hole Interaction Screening

As noted, the effect of including screening in the polarizability calculation in Σ^*scTDHF*^ is to *increase* IE. Since the electron–hole interaction in the polarizability calculation is screened in this case, pole positions, Ω_+_^α^, shift to *larger* values. Comparison of IE from Σ^*TDHF*^ in Tables 2 and S1 to S6 in the Supporting Information show that IE shifts induced by screening are remarkably consistent for particular types of state: *n*, π_1_ and π_2_. Table 9 shows mean shifts and maximum and minimum shifts for these states, on going from Σ^*TDHF*^ to Σ^*scTDHF*^ calculations. Values for π_3_ states are not given in Table 9 as these are associated with shakeup excitations. Shifts associated with screening are small (less than 0.3 eV) and have narrow dispersions, with the largest shifts occurring in nonbonding *n*_*N*_ states in Σ^*scTDHF*^.

**Table 9:** Mean IE Shifts and Range of Shifts for scTDHF Compared to TDHF from Including Screening in the Polarizability, in eV

state	mean shift	shift range
π_1_	0.07	0.04 to 0.15
π_2_	0.13	0.09 to 0.18
*n*_1,2,3_	0.24	0.18 to 0.27

### Effects of Vertex Correction

Inclusion of the vertex term in the Σ^*vTDHF*^ and Σ^*scvTDHF*^ self-energies results in relatively small changes in IE, which depend on the character of the state concerned and are consistent in sign across most of the molecules in the data set. The largest shifts in IE occur for nonbonding N states in triply bonded diacetylene, cyanoacetylene and cyanogen. The IE for these states are increased by the vertex term by between 0.41 and 0.45 eV, leading to a much improved agreement with experimental IE (Table 5). The vertex term shifts the π_1_ IE for C = C bonds downward by between 0.18 eV (in pyrrole and thiophene) and 0.39 eV (in butadiene) in the small molecules and five-membered rings. The downward shift of the π_1_ C = C bond states in the six-membered rings is smaller, ranging from 0.07 eV in benzene to 0.17 eV in pyrazine. Nonbonding O states in acrolein, glyoxal, acetone and furan are shifted upward by the vertex term, with shifts ranging from 0.02 to 0.34 eV (both glyoxal *n*_*O*_ states). Inspection of Table 3 shows that inclusion of the vertex term in the vTDHF or scvTDHF self-energies leads to larger ΔIE values, compared to TDHF or scTDHF, especially for *t*-butadiene. However, Tables 4 to 7 show that adding vertex corrections for *n*_*O*_, *n*_*N*_ and π_1_ and π_2_ states in triply bonded molecules and five and six membered rings improves agreement with experiment (with a reduced MAE and MSE closer to zero).

### Basis Set and Method Dependence of IE

Here we compare values for the first IE in benzene from high level CCSD(T) and IP-EOM-CCSD methods to values obtained in this work, including values extrapolated to the complete basis set (CBS) limit. Table 10 gives values of 9.44 and 9.45 eV for CCSD(T) in the CBS limit from two groups.^75,76^ Deleuze and co-workers found values of 9.32 and 9.36 eV for aug-cc-pVDZ and cc-pVTZ basis sets.^75^ Using a def2-TZVPP,^79^ Caruso and co-workers obtained a CCSD(T) value of 9.29 eV. Lange and Berkelbach^78^ found a value of 9.32 eV using a def2-TZVPPD basis and the IP-EOM-CCSD method. These values are comparable to those reported here. In the aug-cc-pVTZ basis, Σ^*scTDHF*^ yields a value of 9.27 eV which rises to 9.40 eV in the aug-cc-pVQZ basis. Addition of the vertex correction diagram at the bottom of Figure 1 results in a value of 9.20 eV in Σ^*scvTDHF*^. Σ^*RPA*^ values with aug-cc-pVTZ and aug-cc-pVQZ basis sets of 9.52 and 9.66 eV are higher than the complete basis set CCSD(T) value of 9.45 eV, as might be expected from the positive MSE value Δ*E*^*RPA*^ of 0.29 eV for π_1_ levels in Table 7.

**Table 10:** Benzene HOMO IE from CCSD(T), IP-EOM-CCSD, and Methods in This Work, in eV

method	basis set	ionization energy
CCSD(T)^75^	aug-cc-pVDZ	9.32
CCSD(T)^75^	cc-pVTZ	9.36
CCSD(T)^75^	CBS	9.44
CCSD(T)^76^	CBS	9.45
CCSD(T)^77^	def2-TZVPP	9.29
IP-EOM-CCSD^78^	def2-TZVPPD	9.32
scTDHF (this work)	aug-cc-pVTZ	9.27
scTDHF (this work)	aug-cc-pVQZ	9.40
scvTDHF (this work)	aug-cc-pVTZ	9.20
RPA (this work)	aug-cc-pVTZ	9.52
RPA (this work)	aug-cc-pVQZ	9.66

As noted in the Computational Methods Section, improving the basis from aug-cc-pVTZ to aug-cc-pVQZ for *t*-butadiene, benzene and *s*-triazine leads to increases of IE by between 0.1 and 0.2 eV for Σ^*RPA*^, Σ^*TDHF*^ and Σ^*scTDHF*^ and for π, *n*_*N*_ and σ states. Since ΔIE^*RPA*^ values are positive in every case (Tables 3 to 7), this improvement in basis leads to *increased* values of ΔIE^*RPA*^. On the other hand, ΔIE^*TDHF*^ and ΔIE^*scTDHF*^ are mostly less than −0.2 eV so that the improvement in basis leads to *reduced* magnitudes of these IE differences, reinforcing the conclusion that inclusion of the electron–hole attraction kernel in the polarizability is important for accuracy of self-energies using a HF reference state.

## Discussion and Conclusions

As noted in the Introduction, one of the aims of this work is to establish improved methods for treating optical excitations in molecules which are too large to be treated using highly accurate quantum chemical methods. It is recognized that the *G*_0_*W*_0_@HF method yields IE and optical gaps which are larger than those predicted by, e.g. CCSD(T), when the RPA polarizability is used in the screened interaction, *W*_0_. Bruneval and Marques^9^ found a MSE(MAE) for the first IE of a set of 34 molecules^80^ of −0.45(0.46) eV relative to CCSD(T). Here *G*_0_*W*_0_*@HF* overestimates the magnitude of the IE. Similar (but unquantified) results were obtained by Caruso et al.^19^

Knight et al.^12^ applied eight *GW* methods to calculation of first IE and EA for 24 acceptor molecules and compared the results to CCSD(T) calculations. They found that *G*_0_*W*_0_*@HF* yielded a MAE of 0.43 eV for IE of the benchmark set with all IE overestimated (MSE 0.43 eV) while scGW gave a MAE of 0.60 eV with all IE underestimated (MSE −0.60 eV). The best performing starting point for IE calculations of the acceptor molecules was *G*_0_*W*_0_*@LC* - ωPBE. Koval et al.^42^ found that for a set of atoms and small molecules *G*_0_*W*_0_*@HF* gave a MAE compared to CCSD(T) of 0.28 eV in a cc-pVTZ basis.

Thus, MSE/MAE for first IE from *G*_0_*W*_0_*@HF* in the literature typically range from 0.3 to 0.5 eV. The range of MSE/MAE found for *G*_0_*W*_0_*@HF* in this work (denoted Σ^*RPA*^) depends on the orbital character and which IE of that type (first, second, etc.) is considered. MSE/MAE for the highest lying π_1_ levels range from 0.26 eV for alkenes and carbonyl molecules (Table 3), to 0.30 eV for five and six membered ring systems (Table 7) and to 0.40 in triply bonded systems (Table 5). The MSE/MAE for *G*_0_*W*_0_*@HF* for lone pair states is larger. For O lone pairs the MSE/MAE for the first or first and second nonbonding IE is 0.55 eV (Table 4) and for N nonbonding IE in five and six membered rings, the MSE/MAE is 0.82 eV (Table 6). In the triply bonded systems with N nonbonding IE the MSE/MAE is 1.11 eV (Table 5). As far as we know, this systematic variation in errors of *G*_0_*W*_0_*@HF* has not been previously noted. These MSE/MAE in *G*_0_*W*_0_*@HF* depend in turn on the magnitudes of self-energy shifts, ΔIE^*HF*^, required to align HF eigenvalue predictions of IE via Koopmans theorem with experimental IE values and are reasonably transferable between molecules for particular bonding types.

It is clear from Figures 2 and 4 to 6 that Σ^*RPA*^ (*G*_0_*W*_0_*@HF*) consistently overestimates IE. The approach to correcting this which has been widely adopted is to use a DFT or hybrid DFT starting point rather than HF.^7,9,12,18,20,33,35^ Here, instead, we include the electron–hole attraction term in the polarizability (Π^*RPA*^ is replaced by Π^*TDHF*^ or Π^*scTDHF*^). TDHF excitation energies lie several eV below RPA energies and scTDHF energies are intermediate between these two. Typically, Σ^*TDHF*^ is larger than is needed to reduce ΔIE to zero and *overcorrects* ΔIE ^*HF*^ (Black crosses in Figures 2 and 4 to 6). Screening the electron–hole attraction in the polarizability shifts self-energy pole positions to larger energies and reduces these overcorrections (red circles). Adding the vertex correction term illustrated in Figure 2 further reduces ΔIE for all state characters, except π_1_ states (blue triangles and green diamonds).

*G*_0_*W*_0_*@HF* self-energies are too small because RPA polarizability pole positions lie at energies which are too large. Corresponding pole positions in self-energies are the sum of a HF eigenvalue, ε^*HF*^, and an RPA, TDHF or scTDHF energy (Ω_+_^α^, eq 20 to 22). Shifting self-energy pole positions to lower energy magnitudes increases the self-energy in the energy window where the QP equation (eq 23) is satisfied for valence states (Figure 2).

Lewis and Berkelbach^34^ applied a *GW* method to calculation of first IE of 20 of the smallest systems in the *GW*100 test set. They used a polarizability from EOM-CCSD and found that the MSE(MAE) increased from 0.19(0.31) eV (*G*_0_*W*_0_*@HF*) to −0.31(0.52) eV when a coupled cluster screened interaction (*G*_0_*W*_*CC*_*@HF*) was used. Iterating the eigenvalues of the Green’s function in addition (*G*_*ev*_*W*_*CC*_*@HF*) resulted in a further increase in error magnitudes to −0.44(0.64) eV. These observations can be reconciled with results presented in this work: excitation energies from EOM-CCSD calculations are expected to lie below those from TDHF or scTDHF and therefore corresponding self-energy pole positions lie at lower energies than those of Σ^*TDHF*^ and a greater overcorrection of HF eigenvalue estimates of IE. In nonself-consistent solutions of Hedin’s equations, an approximation is needed which consistently yields self-energy pole positions in approximately the right places.

Recent work by Bruneval and Förster^20^ found that for the *GW*100 database^3^ the MAD for *G*_0_*W*_0_*@HF* of 0.30 eV increased to 0.49 eV when a vertex correction term expanded in the screened interaction and denoted *G*3*W*2 was added to the self-energy. These mean vertex correction shifts (0.19 eV) are of a similar magnitude to those found in this work. Maggio and Kresse^13^ found large vertex corrections for the HOMO of dinitrogen and ammonia which are lone pair N states. They found vertex correction shifts of 0.61 and 0.51 eV, respectively, which compare to the value of 0.41 eV for the *n*_*N*1_ state in cyanogen in this work. However, they did not find large negative shifts for the HOMOs of acetylene and ethylene, which might be expected from the negative shifts for π_1_ states in this work.

One success of the scTDHF approach that is described here is that it predicts that shakeup photoionization of π_3_ states in the six π electron systems and π_2_ state in *t*-butadiene studied here, in agreement with experiment and ADC(3) calculations.^57,60^ QP weights for these states are significantly less than QP weights for other states in scTDHF, but remain around 0.9 in RPA. This reduction of QP weight and multiple roots of the QP equation, eq 23, is an indication of a breakdown of the single particle picture of photionization. Pole positions in the RPA self-energy are too large in magnitude and lead to single roots of the QP equation instead.

Self-energies from RPA and scTDHF methods have been used to calculate the optical absorption spectrum in BSE-TDA calculations on C_60_.^53^ There it was found that agreement with the experimental optical absorption, in terms of peak positions and intensities, was significantly improved on going from self-energies from RPA to scTDHF.

## Data Availability

The data that support the findings of this study are available from the corresponding author upon reasonable request. The Exciton code used in this work is available from CHP under a Mozilla Public License 2.0 on reasonable request, via e-mail to Charles.Patterson@tcd.ie.

## Supporting Information Available

A derivation of the inverse of the polarizability from TDHF theory; tables which present IE data from experiment and the five methods for calculating IE used in this work, namely HF, RPA, TDHF, scTDHF, vTDHF, and scvTDHF; a comparison of IE for *t*-butadiene, benzene, and *s*-triazine from RPA, TDHF, and scTDHF self-energies using the aug-cc-pVTZ and aug-cc-pVQZ basis sets (PDF)
