# Correction to Molecular Ionization Energies from GW and Hartree–Fock Theory: Polarizability, Screening and Self-Energy Vertex Corrections

**DOI:** 10.1021/acs.jctc.4c01274

**Published:** 2024-10-12

**Authors:** Charles H. Patterson

**Affiliations:** School of Physics, Trinity College Dublin, Dublin D02 PN40, Ireland

In our article,^1^ eqs 20 to 22 should contain a double sum over indices *i*, *a* and *i*′, *a*′ and not a single sum over *a*, *i*, as originally shown. Also the indices *i* and *a* in the factors ⟨*na*∥*ji*⟩ in the second terms of these equations were exchanged. The corrected equations are
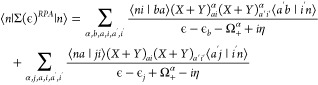
1
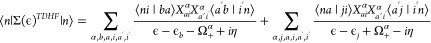
2
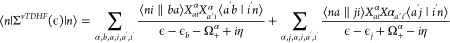
3

The code used in the original article^1^ is consistent with eqs 1 to 3 above, and the results of the work are not affected by these errors.
