# Supplementary material for: Molecular Ionization Energies from GW and Hartree–Fock Theory: Polarizability, Screening, and Self-Energy Vertex Corrections
Source: J Chem Theory Comput. 2024 Aug 27;20(17):7479–93. doi: 10.1021/acs.jctc.4c00795 (PMC11391582; doi:10.1021/acs.jctc.4c00795)
Supplement: Supplementary file 1 — ct4c00795_si_001.pdf [file ct4c00795_si_001.pdf]

# Supporting Information for Molecular ionization energies from $GW$ and Hartree-Fock theory: polarizability, screening and self-energy vertex corrections

Charles H. Patterson*

*School of Physics, Trinity College Dublin, Dublin 2, D02 PN40, Ireland*

E-mail: Charles.Patterson@tcd.ie

**Abstract**

This document contains:

- Derivation of the TDHF polarizability (Eq. 15 in the main text).
- Experimental and computed ionization potentials for molecules considered in this work in eV. Column headings RPA, TDHF, scTDHF refer to the type of polarizability calculation used to calculate the self-energy. The letter v in the polarizability heading indicates that vertex corrections are included in the self-energy.
- Comparison of IE for *t*-butadiene, benzene and *s*-triazine from $\Sigma^{RPA}$, $\Sigma^{TDHF}$ and $\Sigma^{scTDHF}$ using aug-cc-pVTZ and aug-cc-pVQZ basis sets.

References to sources of experimental ionization energies from photoemission data are given at the end of the document.

# Spectral Representation of TDHF Polarizability $\Pi^{TDHF}$

The inverse of $\mathbf{C}\omega - \mathbf{H}$ is,

$$\begin{pmatrix} \mathbf{X} & \mathbf{Y}^* \\ \mathbf{Y} & \mathbf{X}^* \end{pmatrix} \begin{pmatrix} \omega - \mathbf{\Omega}^o_+ + i\eta\mathbf{1} & \mathbf{0} \\ \mathbf{0} & \omega + \mathbf{\Omega}^o_+ - i\eta\mathbf{1} \end{pmatrix}^{-1} \begin{pmatrix} \mathbf{X}^* & \mathbf{Y}^* \\ -\mathbf{Y} & -\mathbf{X} \end{pmatrix}. \tag{1}$$

where,

$$\mathbf{H} = \begin{pmatrix} \mathbf{A} & \mathbf{B} \\ \mathbf{B}^* & \mathbf{A}* \end{pmatrix} \tag{2}$$

and

$$\mathbf{C} = \begin{pmatrix} \mathbf{1} & \mathbf{0} \\ \mathbf{0} & -\mathbf{1} \end{pmatrix} \tag{3}$$

# Inverse of BSE Hamiltonian

$$\begin{pmatrix} \mathbf{A} & \mathbf{B} \\ \mathbf{B}^* & \mathbf{A}^* \end{pmatrix} \begin{pmatrix} \mathbf{X} & \mathbf{Y}^* \\ \mathbf{Y} & \mathbf{X}^* \end{pmatrix} = \begin{pmatrix} \mathbf{1} & \mathbf{0} \\ \mathbf{0} & -\mathbf{1} \end{pmatrix} \begin{pmatrix} \mathbf{X} & \mathbf{Y}^* \\ \mathbf{Y} & \mathbf{X}^* \end{pmatrix} \begin{pmatrix} \mathbf{\Omega} & \mathbf{0} \\ \mathbf{0} & -\mathbf{\Omega} \end{pmatrix} \tag{4}$$

These equations may be abbreviated as $\mathbf{H}\xi = \mathbf{C}\xi\mathbf{\Omega}$ in an obvious notation. The matrix,

$$\xi = \begin{pmatrix} \mathbf{X} & \mathbf{Y}^* \\ \mathbf{Y} & \mathbf{X}^* \end{pmatrix}, \tag{5}$$

contains first order density matrix amplitudes: $X_{ia}$ and $Y_{ia}$. In the absence of an external perturbation, the homogeneous equations,

$$\mathbf{H}\xi^o = \mathbf{C}\xi^o\mathbf{\Omega}^o, \tag{6}$$

are a pair of generalized eigenvalue problems,

$$\mathbf{H}\begin{pmatrix}\mathbf{X}^{\alpha}\\ \mathbf{Y}^{\alpha}\end{pmatrix} = \mathbf{C}\begin{pmatrix}\mathbf{X}^{\alpha}\\ \mathbf{Y}^{\alpha}\end{pmatrix}\Omega^{\alpha}_{+},$$

$$\mathbf{H}\begin{pmatrix}\mathbf{Y}^{*,\alpha}\\ \mathbf{X}^{*,\alpha}\end{pmatrix} = \mathbf{C}\begin{pmatrix}\mathbf{Y}^{*,\alpha}\\ \mathbf{X}^{*,\alpha}\end{pmatrix}\Omega^{\alpha}_{-},$$

where eigenvalues (labelled $\alpha$) are in positive and negative pairs, where $\Omega^{\alpha}_{-} = -\Omega^{\alpha}_{+}$, and eigenvectors are chosen to have the norm condition,

$$\mathbf{X}^{*\alpha}\mathbf{X}^{\beta} - \mathbf{Y}^{*\alpha}\mathbf{Y}^{\beta} = \pm\delta_{\alpha\beta}, \tag{7}$$

where the sign of $\pm$ is the sign of the eigenvalue, $\Omega^{\alpha}_{\pm}$. $\mathbf{H}$ can therefore be expressed as $\mathbf{H} = \mathbf{C}\xi^{o}\mathbf{\Omega}^{o}\xi^{o,-1}$ with inverse,

$$\mathbf{H}^{-1} = \xi^{o}\mathbf{\Omega}^{o}\xi^{o,-1}\mathbf{C}^{-1}. \tag{8}$$

The inverse $(\mathbf{C}\omega - \mathbf{H})^{-1}$ is $\xi^{o}(\omega - \mathbf{\Omega}^{o})^{-1}\xi^{o,-1}\mathbf{C}^{-1}$, where $\omega$ is a diagonal matrix with the dimension of the $\mathbf{A}$ matrix and contains the frequency of any perturbing potential. The inverse of $\mathbf{C}\omega - \mathbf{H}$ is,

$$\begin{pmatrix}\mathbf{X} & \mathbf{Y}^{*}\\ \mathbf{Y} & \mathbf{X}^{*}\end{pmatrix}\begin{pmatrix}\omega - \mathbf{\Omega}^{o}_{+} + i\eta\mathbf{1} & \mathbf{0}\\ \mathbf{0} & \omega + \mathbf{\Omega}^{o}_{+} - i\eta\mathbf{1}\end{pmatrix}^{-1}\begin{pmatrix}\mathbf{X}^{*} & \mathbf{Y}^{*}\\ -\mathbf{Y} & -\mathbf{X}\end{pmatrix}. \tag{9}$$

Sign pairing of eigenvalues has been used to rewrite the bottom right block of the inverse in Eq. 9, -$(\mathbf{\Omega}^{o})_{-}$, as $+(\mathbf{\Omega}^{o})_{+}$. Infinitesimal imaginary parts have been added to shift the poles off the real axis. The product $\xi^{o,-1}\mathbf{C}$ is,

$$\begin{pmatrix}\mathbf{X}^{*} & -\mathbf{Y}^{*}\\ -\mathbf{Y} & \mathbf{X}\end{pmatrix}\begin{pmatrix}\mathbf{1} & \mathbf{0}\\ \mathbf{0} & -\mathbf{1}\end{pmatrix} = \begin{pmatrix}\mathbf{X}^{*} & \mathbf{Y}^{*}\\ -\mathbf{Y} & -\mathbf{X}\end{pmatrix}. \tag{10}$$

Explicit evaluation of the inverse in Eq. 9 yields the density-density response matrix,

$$\begin{pmatrix} \mathbf{X} & \mathbf{Y}^* \\ \mathbf{Y} & \mathbf{X}^* \end{pmatrix} = \left[ \begin{pmatrix} \frac{\mathbf{XX}^*}{\omega\mathbf{1}-\mathbf{\Omega}_\alpha^+ + i\eta\mathbf{1}} & \frac{\mathbf{XY}^*}{\omega\mathbf{1}-\mathbf{\Omega}_\alpha^+ + i\eta\mathbf{1}} \\ \frac{\mathbf{YX}^*}{\omega\mathbf{1}-\mathbf{\Omega}_\alpha^+ + i\eta\mathbf{1}} & \frac{\mathbf{YY}^*}{\omega\mathbf{1}-\mathbf{\Omega}_\alpha^+ + i\eta\mathbf{1}} \end{pmatrix} - \begin{pmatrix} \frac{\mathbf{Y}^*\mathbf{Y}}{\omega\mathbf{1}+\mathbf{\Omega}_\alpha^+ - i\eta\mathbf{1}} & \frac{\mathbf{Y}^*\mathbf{X}}{\omega\mathbf{1}+\mathbf{\Omega}_\alpha^+ - i\eta\mathbf{1}} \\ \frac{\mathbf{X}^*\mathbf{Y}}{\omega\mathbf{1}+\mathbf{\Omega}_\alpha^+ - i\eta\mathbf{1}} & \frac{\mathbf{X}^*\mathbf{X}}{\omega\mathbf{1}+\mathbf{\Omega}_\alpha^+ - i\eta\mathbf{1}} \end{pmatrix} \right] \begin{pmatrix} \mathbf{V} & \mathbf{V}^* \\ \mathbf{V} & \mathbf{V}^* \end{pmatrix} \tag{11}$$

The top row of the matrix is the first order density matrix element, $\mathbf{P}_{ai}$, and the second row is $\mathbf{P}_{ia}$. In the TDA $Y$ is zero and the sole contribution to the resonant term in the polarizability is $\mathbf{XX}^*$ while the sole contribution to the anti-resonant term is $\mathbf{X}^*\mathbf{X}$. The former is the retarded polarizability which is accompanied by the particle part of the self-energy while the latter is the advanced polarizability which is accompanied by the hole part of the self energy. For molecules with real Orbitals and real valued $\mathbf{X}$ and $\mathbf{Y}$ vectors, Orbital products $\phi_a^*(r)\phi_i(r)$ and $\phi_i^*(r)\phi_a(r)$ are equivalent and so the 2 x 2 blocked polarizability reduces to

$$(\mathbf{X}+\mathbf{Y}) \left[ \left(\omega - \mathbf{\Omega}_+^\alpha + i\eta\mathbf{1}\right)^{-1} - \left(\omega + \mathbf{\Omega}_+^\alpha - i\eta\mathbf{1}\right)^{-1} \right] (\mathbf{X}+\mathbf{Y})^T \tag{12}$$

The RPA equations are commonly solved in a way which takes advantage of the fact that A - B is diagonal.

$$\begin{pmatrix} \mathbf{X} \\ \mathbf{Y} \end{pmatrix} \left(\omega - \mathbf{\Omega}_+^\alpha + i\eta\mathbf{1}\right)^{-1} \begin{pmatrix} \mathbf{X}^* & \mathbf{Y}^* \end{pmatrix} - \begin{pmatrix} \mathbf{Y}^* \\ \mathbf{X}^* \end{pmatrix} \left(\omega + \mathbf{\Omega}_-^\alpha - i\eta\mathbf{1}\right)^{-1} \begin{pmatrix} \mathbf{Y} & \mathbf{X} \end{pmatrix} \tag{13}$$

where the particular approximation depends on the $\mathbf{A}$ and $\mathbf{B}$ matrices in Table 1 in the main text.

Table S1: Ionization energies in eV for $t$-butadiene, acrolein, glyoxal and acetone

| Orbital | Symmetry | Expt | HF | RPA | TDHF | scTDHF | vTDHF | scvTDHF |
|---|---|---|---|---|---|---|---|---|
| $t$-butadiene[1] | $C_{2v}$ | | | | | | | |
| $\pi_1$ | $b_g$ | 9.07 | 8.80 | 9.30 | 9.20 | 9.24 | 8.79 | 8.85 |
| $\pi_2$ | $a_u$ | 11.48 | 12.11 | 12.05 | 11.29 | 11.44 | 11.45 | 11.58 |
| | $a_g$ | 12.2 | 13.52 | 13.17 | 12.28 | 12.45 | 12.05 | 12.20 |
| | $b_u$ | 13.49 | 14.87 | 14.42 | 13.41 | 13.60 | 13.30 | 13.47 |
| | $a_g$ | 13.9 | 15.19 | 14.54 | 13.49 | 13.63 | | |
| | $a_g$ | 15.3 | 17.34 | 16.52 | 15.04 | 15.34 | | |
| | $b_u$ | 15.8 | 17.57 | 16.70 | 15.30 | 15.57 | | |
| acrolein[2] | $C_s$ | | | | | | | |
| $n_O$ | $a'$ | 10.10 | 11.79 | 10.81 | 9.36 | 9.61 | 9.51 | 9.73 |
| $\pi_{CC}$ | $a''$ | 10.92 | 10.94 | 11.29 | 11.02 | 11.10 | 10.77 | 10.85 |
| $\pi_{CO}$ | $a''$ | 13.7 | 14.79 | 14.38 | 13.32 | 13.52 | 13.61 | 13.77 |
| | $a'$ | 13.7 | 15.31 | 14.82 | 13.82 | 13.97 | | |
| | $a'$ | 14.6 | 16.51 | 15.61 | 14.31 | 14.54 | 14.38 | 14.57 |
| | $a'$ | 16.05 | 18.21 | 17.18 | 15.48 | 15.83 | | |
| glyoxal[3] | $C_{2h}$ | | | | | | | |
| $n_{O1}$ | $a_g$ | 10.6 | 12.07 | 11.35 | 10.10 | 10.31 | 10.15 | 10.33 |
| $n_{O2}$ | $b_u$ | 12.19 | 14.35 | 13.06 | 11.35 | 11.62 | 11.72 | 11.96 |
| $\pi_{CO1}$ | $b_g$ | 14.0 | 14.52 | 14.44 | 13.69 | 13.88 | 13.72 | 13.86 |
| $\pi_{CO2}$ | $a_u$ | 15.4 | 16.25 | 16.05 | 15.28 | 15.38 | 15.43 | 15.52 |
| acetone[4] | $C_{2v}$ | | | | | | | |
| $n_O$ | $b_2$ | 9.71 | 11.31 | 10.39 | 9.00 | 9.24 | 9.14 | 9.35 |
| $\pi_{CO}$ | $b_1$ | 12.59 | 13.31 | 13.00 | 12.12 | 12.32 | 12.17 | 12.32 |
| | $b_2$ | 13.40 | 14.70 | 14.35 | 13.50 | 13.66 | 13.39 | 13.54 |
| | $a_2$ | 13.95 | 15.32 | 15.07 | 14.32 | 14.46 | 14.24 | 14.37 |
| | $a_1$ | 15.52 | 15.35 | 14.82 | 13.85 | 14.02 | 13.80 | 13.97 |
| | $b_1$ | 17.75 | 17.07 | 16.63 | 15.75 | 15.87 | 15.86 | 15.98 |

Table S2: Ionization energies in eV for diacetylene, cyanoacetylene and cyanogen

| Orbital | Symmetry | Expt | HF | RPA | TDHF | scTDHF | vTDHF | scvTDHF |
|---|---|---|---|---|---|---|---|---|
| diacetylene[5] | $D_{\infty h}$ | | | | | | | |
| $\pi_1$ | $\pi_g$ | 10.17 | 10.07 | 10.47 | 10.37 | 10.41 | 9.98 | 10.03 |
| $\pi_2$ | $\pi_u$ | 12.62 | 13.40 | 13.19 | 12.42 | 12.54 | 12.53 | 12.64 |
| | $\sigma_g$ | 16.61 | 19.22 | 18.23 | 16.89 | 17.09 | 16.99 | 17.16 |
| cyanoacetylene[5] | $C_{\infty v}$ | | | | | | | |
| $\pi_1$ | $\pi$ | 11.60 | 11.67 | 12.03 | 11.90 | 11.95 | 11.55 | 11.60 |
| $n_N$ | $\sigma^+$ | 13.54 | 16.12 | 14.63 | 12.76 | 13.03 | 13.23 | 13.45 |
| $\pi_2$ | $\pi$ | 14.03 | 14.89 | 14.71 | 13.97 | 14.09 | 14.08 | 14.18 |
| cyanogen[5] | $D_{\infty h}$ | | | | | | | |
| $\pi_1$ | $\pi_g$ | 13.36 | 13.50 | 13.82 | 13.66 | 13.72 | 13.34 | 13.40 |
| $n_{N1}$ | $\sigma_u^+$ | 14.49 | 17.01 | 15.62 | 13.80 | 14.05 | 14.26 | 14.46 |
| $n_{N2}$ | $\sigma_g^+$ | 14.86 | 17.44 | 15.98 | 14.10 | 14.36 | 14.61 | 14.81 |
| $\pi_2$ | $\pi_u$ | 15.47 | 16.33 | 16.23 | 15.59 | 15.70 | 15.67 | 15.76 |

Table S3: Ionization energies in eV for isobutene and methylenecyclopropene (MCP)

| Orbital | Symmetry | Expt | HF | RPA | TDHF | scTDHF | vTDHF | scvTDHF |
|---|---|---|---|---|---|---|---|---|
| isobutene[6] | $C_{2v}$ | | | | | | | |
| $\pi_1$ | $b_1$ | 9.41 | 9.42 | 9.68 | 9.42 | 9.49 | 9.12 | 9.20 |
| | $b_2$ | 11.86 | 13.06 | 12.04 | 11.74 | 11.90 | 11.60 | 11.77 |
| | $a_1$ | 12.84 | 14.01 | 13.51 | 12.63 | 12.79 | 12.56 | 12.73 |
| | $b_2$ | 13.2 | 14.19 | 13.81 | 12.99 | 13.15 | 12.92 | 13.09 |
| | $a_2$ | 13.6 | 14.64 | 14.25 | 13.41 | 13.57 | 13.41 | 13.57 |
| | $b_1$ | 14.95 | 16.11 | 15.56 | 14.60 | 14.78 | 14.75 | 14.93 |
| | $a_1$ | 15.2 | 16.61 | 15.91 | 14.87 | 15.05 | 14.98 | 15.16 |
| | $b_2$ | 15.2 | 16.87 | 16.11 | 15.26 | 15.43 | 15.36 | 15.44 |
| MCP[7] | $C_{2v}$ | | | | | | | |
| $\pi_1$ | $b_2$ | 8.41 | 8.08 | 8.51 | 8.31 | 8.37 | 7.99 | 8.05 |
| | $b_1$ | 10.95 | 12.01 | 11.83 | 11.08 | 11.22 | 10.83 | 10.96 |
| $\pi_2$ | $b_2$ | 13.3 | 13.87 | 13.71 | 12.98 | 13.04 | 13.09 | 13.18 |
| | $a_1$ | 12.9 | 14.35 | 13.82 | 12.86 | 13.04 | 12.77 | 12.94 |
| | $b_1$ | 14.3 | 15.86 | 15.21 | 14.15 | 14.25 | 14.10 | 14.30 |
| | $a_1$ | 15.9 | 17.84 | 16.87 | 15.48 | 15.69 | | |

Table S4: Ionization energies in eV for five membered rings. Two values are shown when the QP equation solution lies adjacent to a pole in the self-energy.

| Orbital | Symmetry | Expt | HF | RPA | TDHF | scTDHF | vTDHF | scvTDHF |
|---|---|---|---|---|---|---|---|---|
| cyclopentadiene[8] | | | | | | | | |
| | $C_{2v}$ | | | | | | | |
| $\pi_1$ | $a_2$ | 8.56 | 8.34 | 8.79 | 8.65 | 8.69 | 8.31 | 8.37 |
| $\pi_2$ | $b_1$ | 10.72 | 11.25 | 11.27 | 10.68 | 10.80 | 10.64 | 10.74 |
| | $b_2$ | 12.2 | 13.68 | 13.21 | 12.22 | 12.40 | 12.07 | 12.24 |
| | $a_1$ | 12.6 | 13.82 | 13.31 | 12.32 | 12.50 | 12.17 | 12.34 |
| | $a_1$ | 13.2 | 14.35 | 13.72 | 12.66 | 12.85 | 12.58 | 12.76 |
| | $b_2$ | 13.8 | 15.25 | 14.56 | 13.47 | 13.67 | 13.39 | 13.58 |
| $\pi_3$ | $b_1$ | 15.0 | 16.16 | 15.49 | 13.46/14.75 | 14.15/15.32 | | |
| furan[9] | | | | | | | | |
| | $C_{2v}$ | | | | | | | |
| $\pi_1$ | $a_1$ | 8.83 | 8.73 | 9.20 | 9.03 | 9.08 | 8.78 | 8.84 |
| $\pi_2$ | $b_1$ | 10.39 | 10.87 | 10.71 | 9.97 | 10.12 | 10.09 | 10.22 |
| | $a_1$ | 12.96 | 14.73 | 13.96 | 12.80 | 12.99 | 12.80 | 12.97 |
| $n_O$ | $a_1$ | 13.86 | 15.41 | 14.44 | 13.11 | 13.34 | 13.17 | 13.40 |
| | $b_2$ | 14.51 | 15.73 | 15.10 | 14.08 | 14.26 | 13.99 | 14.17 |
| | $b_2$ | 15.26 | 16.60 | 15.95 | 14.85 | 15.05 | 14.77 | 14.96 |
| $\pi_3$ | $b_1$ | 15.26 | 17.24 | 15.64 | 13.31/15.32 | 13.76 | | |
| imidazole[10] | | | | | | | | |
| | $C_s$ | | | | | | | |
| $\pi_1$ | $a''$ | 8.78 | 8.77 | 9.20 | 8.99 | 9.05 | 8.79 | 8.84 |
| $\pi_2$ | $a''$ | 10.3 | 10.92 | 10.72 | 9.87 | 10.05 | 10.07 | 10.19 |
| $n_N$ | $a'$ | 10.3 | 11.92 | 10.89 | 9.44 | 9.70 | 9.68 | 9.89 |
| | $a'$ | 13.7 | 15.56 | 14.93 | 13.91 | 14.08 | 13.77 | 13.94 |
| $\pi_3$ | $a''$ | 14.0 | 16.43 | 15.81 | 13.25 | 13.59 | | |
| | $a'$ | 14.7 | 16.33 | 15.17 | 14.73 | 14.94 | | |
| | $a'$ | 15.3 | 16.62 | 15.80 | 14.58 | 14.84 | | |
| pyrrole[9] | | | | | | | | |
| | $C_{2v}$ | | | | | | | |
| $\pi_1$ | $a_2$ | 8.02 | 8.12 | 8.53 | 8.28 | 8.35 | 8.11 | 8.17 |
| $\pi_2$ | $b_1$ | 9.05 | 9.44 | 9.44 | 8.78 | 8.92 | 8.88 | 8.98 |
| | $a_1$ | 12.85 | 14.36 | 13.73 | 12.67 | 12.85 | 12.58 | 12.75 |
| $\pi_3$ | $b_1$ | 12.85 | 15.47 | 14.28 | 12.27 | 12.65 | | |
| | $b_2$ | 13.57 | 14.92 | 14.27 | 13.25 | 13.57 | 13.15 | 13.41 |
| | $a_1$ | 14.27 | 16.13 | 15.37 | 14.21 | 14.42 | | |
| | $b_2$ | 14.80 | 15.88 | 15.23 | 14.18 | 14.33 | 14.09 | 14.21 |
| thiophene[9] | | | | | | | | |
| | $C_{2v}$ | | | | | | | |
| $\pi_1$ | $a_2$ | 8.85 | 8.93 | 9.26 | 8.96 | 9.04 | 8.80 | 8.86 |
| $\pi_2$ | $b_1$ | 9.49 | 9.44 | 9.55 | 9.10 | 9.21 | 9.11 | 9.18 |
| $n_S$ | $a_1$ | 12.00 | 12.93 | 12.50 | 11.67 | 11.82 | 11.63 | 11.76 |
| $\pi_3$ | $b_1$ | 12.46 | 14.23 | 13.43 | 11.98 | 12.16 | | |
| | $b_2$ | 13.11 | 14.36 | 13.91 | 13.03 | 13.20 | 12.94 | 13.08 |
| | $a_1$ | 13.80 | 15.04 | 14.31 | 13.16 | 13.35 | 13.10 | 13.27 |
| | $b_2$ | 14.23 | 15.70 | 14.96 | 13.76 | 14.03 | | |

Table S5: Ionization energies in eV for six membered rings: benzene, pyridine, *s*-triazine and *s*-tetrazine

| Orbital | Symmetry | Expt | HF | RPA | TDHF | scTDHF | vTDHF | scvTDHF |
|---|---|---|---|---|---|---|---|---|
| benzene[11] | $D_{6h}$ | | | | | | | |
| $\pi_{1,2}$ | $e_{1g}$ | 9.29 | 9.17 | 9.52 | 9.18 | 9.27 | 9.14 | 9.20 |
| | $e_{2g}$ | 11.62 | 13.47 | 12.91 | 11.92 | 12.09 | 11.72 | 11.88 |
| $\pi_3$ | $a_{2u}$ | 12.13 | 13.68 | 12.97 | 11.46 | 11.73 | | |
| | $e_{1u}$ | 13.82 | 16.01 | 15.19 | 13.92 | 14.15 | | |
| | $b_{2u}$ | 14.59 | 16.86 | 15.68 | 14.12 | 14.37 | | |
| | $b_{1u}$ | 15.32 | 17.53 | 16.56 | 15.14 | 15.42 | | |
| pyridine[12] | $C_{2v}$ | | | | | | | |
| $n_N$ | $a_1$ | 9.67 | 11.41 | 10.47 | 9.11 | 9.34 | 9.20 | 9.40 |
| $\pi_1$ | $a_2$ | 9.85 | 9.48 | 9.91 | 9.64 | 9.72 | 9.55 | 9.61 |
| $\pi_2$ | $b_1$ | 10.52 | 10.47 | 10.67 | 10.17 | 10.29 | 10.20 | 10.31 |
| | $b_2$ | 12.60 | 14.17 | 13.62 | 12.63 | 12.80 | 12.43 | 12.59 |
| $\pi_3$ | $b_1$ | 13.19 | 14.76 | 13.96 | 12.35 | 12.62 | | |
| | $a_1$ | 13.79 | 15.71 | 14.79 | 13.38 | 13.71 | | |
| | $b_2$ | 14.51 | 16.30 | 15.52 | 14.27 | 14.56 | | |
| | $a_1$ | 15.6 | 17.82 | 16.88 | 15.47 | 15.74 | | |
| | $b_2$ | 15.8 | 18.01 | 16.78 | 15.12 | 15.43 | | |
| *s*-triazine[12] | $D_{3h}$ | | | | | | | |
| $n_{N1,2}$ | $e'$ | 10.40 | 12.04 | 11.13 | 9.77 | 10.02 | 9.91 | 10.13 |
| $\pi_{1,2}$ | $e''$ | 11.79 | 12.01 | 12.13 | 11.55 | 11.70 | 11.63 | 11.74 |
| $n_{N3}$ | $a'_1$ | 13.37 | 15.76 | 14.26 | 12.35 | 12.67 | | |
| $\pi_3$ | $a'_2$ | 14.64 | 16.70 | 15.87 | 14.29 | 14.55 | | |
| | $e'$ | 14.99 | 16.79 | 16.03 | 14.88 | 15.06 | | |
| *s*-tetrazine[13,14] | $D_{2h}$ | | | | | | | |
| $n_{N1}$ | $b_{3g}$ | 9.72 | 11.45 | 10.55 | 9.20 | 9.42 | 9.28 | 9.46 |
| $\pi_1$ | $b_{2g}$ | 12.0 | 11.82 | 12.32 | 12.09 | 12.18 | 11.99 | 12.05 |
| $n_{N2}$ | $b_{1u}$ | 12.0 | 14.55 | 13.13 | 11.24 | 11.54 | | |
| $n_{N3}$ | $b_{2u}$ | 12.78 | 15.41 | 14.03 | 12.19 | 12.50 | | |
| $n_{N4}$ | $a_g$ | 13.36 | 14.39 | 13.65 | 12.46 | 12.67 | 12.44 | 12.61 |
| $\pi_2$ | $b_{1g}$ | 13.5 | 13.56 | 13.67 | 13.03 | 13.21 | 13.21 | 13.32 |
| $\pi_3$ | $b_{3u}$ | 15.84 | 17.64 | 16.75 | 14.96 | 15.26 | | |

Table S6: Ionization energies in eV for six membered rings: pyrazine, pyridazine and pyrimidine

| Orbital | Symmetry | Expt | HF | RPA | TDHF | scTDHF | vTDHF | scvTDHF |
|---|---|---|---|---|---|---|---|---|
| pyrazine[12] | $D_{2h}$ | | | | | | | |
| $n_{N1}$ | $a_g$ | 9.61 | 11.29 | 10.47 | 9.21 | 9.43 | 9.24 | 9.43 |
| $\pi_1$ | $b_{1g}$ | 10.20 | 9.81 | 10.34 | 10.17 | 10.24 | 10.02 | 10.07 |
| $n_{N2}$ | $b_{1u}$ | 11.38 | 13.68 | 12.26 | 10.58 | 10.83 | 10.89 | 11.15 |
| $\pi_2$ | $b_{2g}$ | 11.80 | 11.92 | 11.99 | 11.36 | 11.52 | 11.50 | 11.61 |
| | $b_{3g}$ | 13.37 | 15.05 | 14.48 | 13.50 | 13.67 | 13.29 | 13.45 |
| $\pi_3$ | $b_{3u}$ | 13.93 | 15.70 | 14.86 | 13.13 | 13.43 | | |
| | $b_{2u}$ | 14.98 | 16.66 | 15.91 | 14.64 | 14.88 | | |
| | $b_{1u}$ | 16.10 | 18.51 | 17.58 | 16.14 | 16.41 | | |
| pyridazine[12] | $C_{2v}$ | | | | | | | |
| $n_{N1}$ | $b_2$ | 9.27 | 11.11 | 10.06 | 8.61 | 8.85 | 8.74 | 8.97 |
| $\pi_1$ | $a_2$ | 10.61 | 10.48 | 10.86 | 10.54 | 10.63 | 10.48 | 10.55 |
| $\pi_2$ | $b_1$ | 11.2 | 11.04 | 11.31 | 10.87 | 10.99 | 10.90 | 10.99 |
| $n_{N2}$ | $a_1$ | 11.3 | 13.09 | 12.20 | 10.88 | 11.10 | 10.89 | 11.12 |
| $\pi_3$ | $b_1$ | 13.97 | 15.74 | 14.86 | 13.16 | 13.46 | | |
| | $a_1$ | 14.27 | 16.07 | 15.10 | 13.66 | 13.91 | | |
| | $b_2$ | 14.66 | 16.40 | 15.59 | 14.25 | 14.52 | | |
| | $b_2$ | 15.90 | 18.19 | 17.27 | 15.87 | 16.11 | | |
| | $a_1$ | 16.8 | 19.06 | 17.83 | 16.14 | 16.42 | | |
| pyrimidine[12] | $C_{2v}$ | | | | | | | |
| $n_{N1}$ | $b_2$ | 9.69 | 11.37 | 10.46 | 9.11 | 9.35 | 9.21 | 9.41 |
| $\pi_1$ | $b_1$ | 10.50 | 10.32 | 10.71 | 10.41 | 10.50 | 10.34 | 10.40 |
| $n_{N2}$ | $a_1$ | 11.22 | 12.94 | 11.95 | 10.56 | 10.80 | 10.71 | 10.91 |
| $\pi_2$ | $a_2$ | 11.40 | 11.54 | 11.61 | 10.98 | 11.14 | 11.10 | 11.20 |
| $\pi_3$ | $b_1$ | 13.9 | 15.74 | 14.91 | 13.29 | 13.56 | | |
| | $a_1$ | 14.1 | 16.01 | 15.17 | 13.86 | 14.10 | | |
| | $b_2$ | 14.4 | 16.26 | 15.40 | 14.07 | 14.31 | | |
| | $a_1$ | 15.8 | 17.79 | 16.87 | 15.48 | 15.74 | | |
| | $b_2$ | 16.88 | 19.15 | 17.89 | 16.19 | 16.49 | | |

Table S7: Ionization energy dependence on basis set for *t*-butadiene, benzene and *s*-triazine in eV for aug-cc-pVTZ (T) and aug-cc-pVQZ (Q) basis sets. Basis set differences are shown in columns following each method. Changes in HF eigenvalues shown are less than 0.02 eV.

| Orbital | Sym | Expt | HF | RPA(T) | RPA(Q) | $\Delta(QT)$ | TDHF(T) | TDHF(Q) | $\Delta(QT)$ | scTDHF(T) | scTDHF(Q) | $\Delta(QT)$ |
|---|---|---|---|---|---|---|---|---|---|---|---|---|
| *t*-butadiene[1] | $C_{2v}$ | | | | | | | | | | | |
| $\pi_1$ | $b_g$ | 9.07 | 8.80 | 9.30 | 9.44 | 0.14 | 9.20 | 9.34 | 0.14 | 9.24 | 9.38 | 0.14 |
| $\pi_2$ | $a_u$ | 11.48 | 12.11 | 12.05 | 12.18 | 0.13 | 11.29 | 11.39 | 0.10 | 11.44 | 11.56 | 0.12 |
| | $a_g$ | 12.2 | 13.52 | 13.17 | 13.30 | 0.13 | 12.28 | 12.40 | 0.12 | 12.45 | 12.58 | 0.13 |
| | $b_u$ | 13.49 | 14.87 | 14.42 | 14.54 | 0.12 | 13.41 | 13.50 | 0.09 | 13.60 | 13.70 | 0.10 |
| | $a_g$ | 13.9 | 15.19 | 14.54 | 14.70 | 0.16 | 13.49 | 13.61 | 0.12 | 13.63 | 13.81 | 0.18 |
| | $a_g$ | 15.3 | 17.34 | 16.52 | 16.65 | 0.13 | 15.04 | 15.16 | 0.12 | 15.34 | 15.46 | 0.12 |
| | $b_u$ | 15.8 | 17.57 | 16.70 | 16.84 | 0.14 | 15.30 | 15.48 | 0.18 | 15.57 | 15.71 | 0.14 |
| benzene[11] | $D_{6h}$ | | | | | | | | | | | |
| $\pi_{1,2}$ | $e_{1g}$ | 9.29 | 9.17 | 9.52 | 9.66 | 0.14 | 9.18 | 9.31 | 0.13 | 9.27 | 9.40 | 0.13 |
| | $e_{2g}$ | 11.62 | 13.47 | 12.91 | 13.06 | 0.15 | 11.92 | 12.07 | 0.15 | 12.09 | 12.25 | 0.16 |
| $\pi_3$ | $a_{2u}$ | 12.13 | 13.68 | 12.97 | 13.10 | 0.13 | 11.46 | 11.58 | 0.12 | 11.73 | 11.83 | 0.10 |
| | $e_{1u}$ | 13.82 | 16.01 | 15.19 | 15.33 | 0.14 | 13.92 | 14.06 | 0.14 | 14.15 | 14.29 | 0.14 |
| | $b_{2u}$ | 14.59 | 16.86 | 15.68 | 15.85 | 0.17 | 14.12 | 14.29 | 0.17 | 14.37 | 14.55 | 0.18 |
| | $b_{1u}$ | 15.32 | 17.53 | 16.56 | 16.67 | 0.11 | 15.14 | 15.25 | 0.11 | 15.42 | 15.51 | 0.09 |
| *s*-triazine[12] | $D_{3h}$ | | | | | | | | | | | |
| $n_{N1,2}$ | $e'$ | 10.40 | 12.04 | 11.13 | 11.30 | 0.17 | 9.77 | 9.95 | 0.18 | 10.02 | 10.18 | 0.16 |
| $\pi_{1,2}$ | $e''$ | 11.79 | 12.01 | 12.13 | 12.29 | 0.16 | 11.55 | 11.71 | 0.16 | 11.70 | 11.86 | 0.16 |
| $n_{N3}$ | $a'_1$ | 13.37 | 15.76 | 14.26 | 14.41 | 0.15 | 12.35 | 12.46 | 0.11 | 12.67 | 12.81 | 0.14 |
| $\pi_3$ | $a'_2$ | 14.64 | 16.70 | 15.87 | 16.00 | 0.13 | 14.29 | 14.41 | 0.12 | 14.55 | 14.69 | 0.14 |
| | $e'$ | 14.99 | 16.79 | 16.03 | 16.18 | 0.15 | 14.88 | 15.02 | 0.14 | 15.06 | 15.18 | 0.12 |

# References


(1) Holland, D. M. P.; MacDonald, M. A.; Hayes, M. A.; Baltzer, P.; Wannberg, B.; Lundqvist, M.; Karlsson, L.; von Niessen, W. An experimental and theoretical study of the valence shell photoelectron spectrum of butadiene *J. Phys. B: At. Mol. Opt. Phys.* **1996**, *29*, 3091-3107.

(2) Ohno, K.; Okamura, K.; Yamakado, H.; Hoshino, S.; Takami, T.; Yamauchi, M. Penning Ionization of HCHO, $CH_2CH_2$, and $CH_2CHCHO$ by Collision with He*($2^3S$) Metastable Atoms *J. Phys. Chem.* **1995**, *99*, 14247-14253.

(3) Turner, D. W.; amd A. D. Baker, C. B.; Brundle, C. R. *Molecular Photoelectron Spectroscopy: A Handbook of He 584 Angstrom Spectra*; Wiley-Interscience, New York, 1970; p 252.

(4) Young, V. Y.; Cheng, K. L. The photoelectron spectra of halogen substituted acetones *J. Chem. Phys.* **1976**, *65*, 3187-3195.

(5) Baker, C.; Turner, D. W. High resolution molecular photoelectron spectroscopy. III. Acetylenes and aza-acetylenes *Proc. Roy. Soc. A* **1968**, *308*, 19-37.

(6) Wiberg, K. B.; Ellison, G. B.; Wendoloski, J. J.; Brundle, C. R.; Kuebler, N. A. Electronic States of Organic Molecules. 3 Photoelectron Spectra of Cycloalkenes and Methylenecycloalkanes *J. Amer. Chem. Soc.* **1976**, *98*, 7179-7182.

(7) Staley, S. W.; Norden, T. D. Structure and Energetics of $C_4H_4^+$ in the Gas Phase. Photoelectron Spectrum of Methylenecyclopropene *J. Amer. Chem. Soc.* **1988**, *111*, 445-449.

(8) Cradock, S.; Findlay, R. H.; Palmer, M. H. Bonding in methyl- and silyl-cyclopentadiene compounds: A study by photoelectron spectroscopy and *ab initio* molecular-orbital calculations *J. Chem. Soc., Dalton Trans.* **1974**, 1650-1654.

(9) Klasinc, L.; Sabljić, A.; Kluge, G.; Rieger, J.; Scholz, M. Chemistry of excited states. Part 13. Assignment of lowest x-ionizations in photoelectron spectra of thiophen, furan, and pyrrole *J. Chem. Soc., Perkin Trans. II* **1982**, 539-543.

(10) Cradock, S.; Findlay, R. H.; Palmer, M. H. The molecular energy levels of the azoles: a study by photoelectron spectroscopy and *ab initio* calculations *Tetrahedron* **1973**, *29*, 2173-2181.

(11) Liu, S.-Y.; Alnama, K.; Matsumoto, J.; Nishizawa, K.; Kohguchi, H.; Lee, Y.-P.; Suzuki, T. He I Ultraviolet Photoelectron Spectroscopy of Benzene and Pyridine in Supersonic Molecular Beams Using Photoelectron Imaging *J. Phys. Chem. A* **2011**, *115*, 2953-2965.

(12) Kishimoto, N.; Ohno, K. Collision Energy Resolved Penning Ionization Electron Spectroscopy of Azines: Anisotropic Interaction of Azines with He*($2^3$S) Atoms and Assignments of Ionic States *J. Phys. Chem. A* **2000**, *104*, 6940-6950.

(13) Gleiter, R.; Heilbronner, R.; Hornung, V. Photoelectron Spectra of Azabenzenes and Azanaphthalenes: I. Pyridine, diazines, s-triazine and s-tetrazine *Helv. Chim. Acta* **1972**, *55*, 255-274.

(14) Tomasello, P.; Wardermann, W.; von Niessen, W.; Cederbaum, L. S. Valence Ionization Spectra of Disubstituted s-Tetrazines: Strong Correlation Effects Induced by Substitution *J. Amer. Chem. Soc.* **1990**, *112*, 94-102.
